# In Silico Structure-Reactivity Analysis of Lignin-Derived Phenolics: O–H Bond Dissociation Enthalpies, Spin Delocalization, and a Propagator/Terminator Framework for Antifungal-Relevant Activity

**DOI:** 10.1021/acsomega.6c07393

**Published:** 2026-09-08

**Authors:** Samir Mallick

**Affiliations:** Department of Chemistry, 6614 University of Pittsburgh, Pittsburgh, Pennsylvania 15260, United States

## Abstract

Plant phenolics represent a prospective source of renewable antifungal drugs, yet the molecular mechanisms underlying their activity within the structurally varied phenolic components of natural products like wood vinegar remain ambiguous. Nine phenolic compounds from corncob (*Zea mays*) and Areca catechu wood vinegars, encompassing the phenol, guaiacol, syringol, and benzenediol categories were examined by in silico methods. Geometries and global reactivity descriptors were derived using density functional theory at the B3LYP-D3BJ/def2-TZVP/CPCM­(water) level, homolytic O–H bond dissociation enthalpies (BDEs) and phenoxyl-radical spin-density distributions were calculated to evaluate and elucidate radical-scavenging capacity, while consensus lipophilicity (log *P*) was estimated to assess membrane permeability. To establish the reactivity trend on a quantitative and transferable basis, the dataset was expanded to include 12 para-, meta-, and ortho-substituted phenols (totaling 21 compounds), the O–H bond dissociation energy in the para-series exhibits a linear correlation with the Hammett constant σ^+^ (ρ ≈ +8.3 kcal mol^–1^, *r* = 0.987). Computed bond dissociation energies (BDEs) were compared with experimental literature values (phenol, 83.2 vs 86.7 ± 0.7 kcal mol^–1^), validating a consistent B3LYP deviation that neutralizes in the relative assessments upon which the analysis depends. The initial O–H bond dissociation energies for the nine natural compounds varied between 73.7 and 83.2 kcal mol^–1^, with phenol exhibiting the lowest reactivity and the two benzenediols demonstrating the highest reactivity. log *P* and BDE exhibited statistical independence (*r* = 0.15), establishing two orthogonal axes of structure-activity, however the Hirshfeld oxygen spin population of the phenoxyl radical showed a strong correlation with BDE (*r* = 0.911), indicating that spin delocalization is the underlying physical cause of the observed reactivity trend. Docking with *Candida albicans* sterol 14α-demethylase (CYP51, PDB 5TZ1) revealed phenolic affinities ranging from −5.0 to −7.6 kcal mol^–1^, which are inferior to the clinical azole fluconazole (−8.4 kcal mol^–1^) and the native inhibitor VT-1161 (−12.2 kcal mol^–1^), the affinity correlated with log *P* (*r* = −0.76) but not with BDE, suggesting that direct inhibition of CYP51 is at best a secondary factor. The diminished second O–H bond dissociation energies of benzenediols facilitate a quinone redox cascade that, when measured against a peroxyl reference bond, categorizes the phenols into radical-terminating monophenols and redox-cycling propagators. The framework offers a structural justification for the antifungal properties of lignin-derived phenolics at the individual component level and emphasizes the unexploited low-BDE/high-log *P* area as a focus for enhancement.

## Graphical abstract

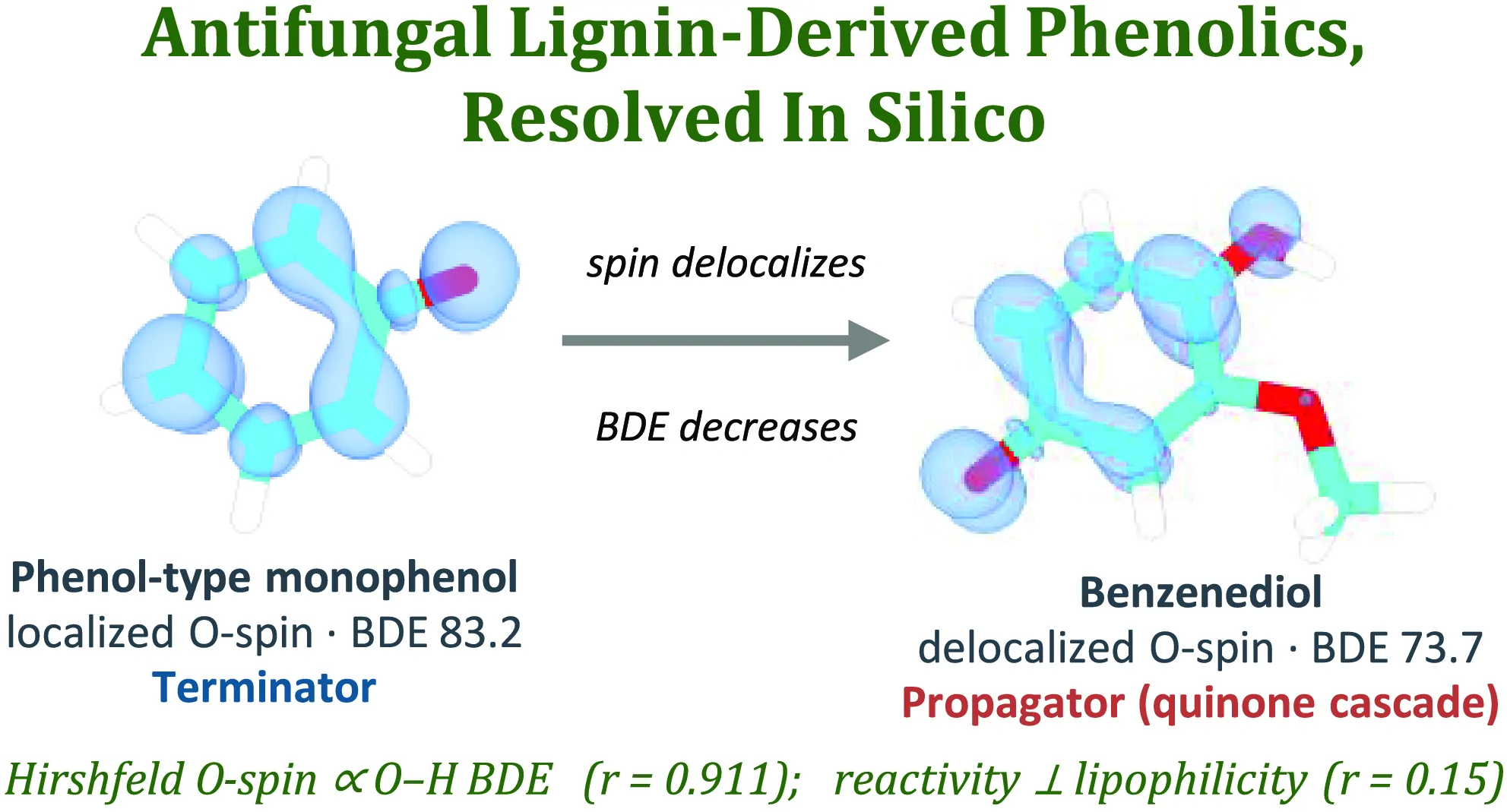

## Introduction

Filamentous fungal infections rank among the most destructive plant ailments, and the rise of antifungal resistance poses a significant risk to human health and global food security.^1,2^ Synthetic fungicides, particularly azoles that obstruct the cytochrome-P450 sterol 14α-demethylase (CYP51) within the ergosterol production pathway of fungi, remain fundamental to chemical control.^3^ The pursuit of antifungal compounds from natural, sustainable sources has accelerated owing to the widespread occurrence of azole resistance and the regulatory and ecological pressures on synthetic agrochemicals. Plant phenolics represent one of the most promising natural options: this category is prevalent in the plant realm, structurally varied, and exhibits recognized antifungal, antibacterial, and antioxidant properties.^4,5^ The chemistry of the phenolic hydroxyl group plays a crucial role in their biological activity. The primary process involves hydrogen-atom transfer (HAT), wherein a propagating radical is quenched through the donation of the phenolic O–H hydrogen. The simplicity of this transfer, evaluated by the homolytic O–H bond dissociation enthalpy (O–H BDE), is the primary factor influencing radical-scavenging effectiveness.^6,7^ Density functional theory has been widely utilized to connect phenolic O–H bond dissociation energies and associated descriptors to antioxidant activity, particularly in methoxyphenols and other lignin-related structures.^31,32^ The reactivity and lipophilicity, the latter governing entry to the fungal membrane, are meticulously adjusted by the quantity and placement of hydroxyl and methoxy groups,^8^ allowing closely related phenols to exhibit significantly different levels of activity. Wood vinegar is the water-based condensate produced during the pyrolysis of lignocellulosic biomass and is rich in cost-effective phenolic compounds. Lignin represents the most plentiful renewable source of aromatic precursors, and its thermochemical transformation produces an intricate mixture of monomeric phenols, chiefly of the guaiacol, syringol, and benzenediol varieties, in addition to organic acids and carbonyl compounds.^9,10^ The antifungal and antioxidant properties of wood vinegars derived from various feedstocks mostly stem from the phenolic fraction rather than the overall acid content.^11–14^

Eleven to 14 Quantitative structure-activity modeling has recently started to link the phenolic structure obtained from lignin to its antibacterial efficacy.^33^ The relationship between phenolic content and activity is stable, although the chemical mechanisms behind this activity remain partially elucidated. The analysis of wood vinegar’s composition is currently conducted using gas chromatography-mass spectrometry, with its activity linked to the overall phenolic fraction or specific key components. These investigations fail to identify which phenols exhibit significant activity, how their substituent arrangements influence reactivity, or the mechanisms of their action issues that are challenging to resolve empirically in a multicomponent mixture but suitable for computational examination. In silico methodologies can offer this mechanistic accuracy at the molecular scale. Frontier-orbital energies, global reactivity indices, the O–H bond dissociation enthalpy, and the spin-density distribution of the resultant phenoxyl radical are electronic-structure parameters derived from density functional theory that collectively assess radical stability and hydrogen-atom-donating capacity.^6^ The prediction of the octanol-water partition coefficient assesses membrane permeability, while molecular docking with validated antifungal target CYP51 ^3^ investigates specific enzyme interactions, monoterpene phenols like thymol and carvacrol have been analyzed against fungal CYP51 and associated targets through docking.^34^ Nine phenolic compounds from the benzenediol, guaiacol, and syringol families were assessed using in silico methods. DFT analyzed their geometries and electronic configurations, O–H bond dissociation enthalpies and radical spin-density distributions were calculated to evaluate radical-scavenging efficacy and clarify its physical basis, lipophilicity was estimated to determine membrane permeability, and molecular docking examined their interaction with *Candida albicans* CYP51. The examination presents a structure-reactivity paradigm for the antifungal properties of lignin-derived phenolics, surpassing their classification as a homogeneous antioxidant reservoir, by categorizing the components and segmenting the phenolic group into two mechanistic categories: radical-terminating monophenols and redox-cycling benzenediol propagators that can initiate a quinone cascade.

## Results

### Geometry Optimization and Validation

All nine compounds were refined to authentic minima on the potential-energy landscape, as evidenced by the lack of imaginary vibrational frequencies. Initially, creosol was located at a torsional saddle point (exhibiting one imaginary mode at −62 cm^–1^) as a result of an eclipsed para-methyl rotor, this was rectified by rotating the methyl group approximately 40° and re-optimizing. In the optimized structures, the intramolecular O–H···O hydrogen bonds in 3-methylcatechol (2.15 Å) and methoxyhydroquinone (2.13 Å) were maintained, aligning with the stabilizing influence of ortho interactions in catechol-type antioxidants.^6^ In the case of open-shell phenoxyl radicals, the spin expectation value ⟨*S* ^2^⟩ consistently approached the optimal doublet value of 0.75 (about 0.77 overall), signifying minimal spin contamination.

### Global Reactivity Descriptors

The energies of frontier orbitals and the computed global reactivity indices, including the HOMO-LUMO gap (Δ*E*), electronegativity (χ), chemical hardness (η), softness (S), and electrophilicity index (ω),^25^ are presented in Table 1. The HOMO energy spans 0.75 eV, from the least effective electron donor (phenol, −6.28 eV) to the most potent (methoxyhydroquinone, −5.53 eV). The LUMO exhibits minimal variation (−0.41 to +0.06 eV) and, with the exception of guaiacylacetone, aligns with the ring π* orbital, hence, the gap, hardness, softness, and electronegativity are closely interconnected, converging toward a singular donor axis. Guaiacylacetone stands out among the LUMO-derived metrics, as its lowest unoccupied orbital corresponds to the side-chain carbonyl π*, while solely its HOMO is comparable to that of the other compounds. Consequently, the frontier descriptors monitor donor potency along a singular correlated axis but fail to distinguish the antifungal-relevant reactivity variations within this group, necessitating the O–H bond dissociation enthalpy as the functional descriptor.

**Table 1:** Frontier-Orbital Energies and Global Reactivity Descriptors of the Nine Phenolics (B3LYP-D3BJ/def2-TZVP/CPCM­(Water), All Values in eV Except Softness S in ev^–1^), Sorted by Increasing First O–H BDE^a^

compound	*E* (HOMO)	*E* (LUMO)	Δ*E*	χ	η	*S*	ω
Methoxyhydroquinone	–5.528	–0.194	5.333	2.861	2.667	0.375	1.535
3-Methylcatechol	–5.917	–0.100	5.817	3.008	2.908	0.344	1.556
Syringol	–5.758	+0.062	5.820	2.848	2.910	0.344	1.394
Creosol	–5.741	–0.174	5.567	2.957	2.784	0.359	1.571
4-Ethylguaiacol	–5.733	–0.135	5.598	2.934	2.799	0.357	1.537
2,4-Di-tert-butylphenol	–5.920	–0.197	5.724	3.059	2.862	0.349	1.634
Guaiacol	–5.857	–0.156	5.702	3.007	2.851	0.351	1.585
Guaiacylacetone	–5.824	–0.743	5.081	3.283	2.541	0.394	2.122
Phenol	–6.275	–0.409	5.866	3.342	2.933	0.341	1.904

a The LUMO of guaiacylacetone is the side-chain carbonyl π*, quantities derived from its LUMO are not comparable with those from the ring-π* of the other compounds.

### O–H Bond Dissociation Enthalpies

In the current HAT mechanism, the O–H bond dissociation energy (BDE) dictates radical-scavenging efficacy: a diminished BDE correlates with an increased propensity for the phenol to give its hydrogen atom.^6,7^ The BDE ladder (Table 2) spans from 73.7 to 83.2 kcal mol^–1^. Phenol establishes the inert upper threshold at 83.2 kcal mol^–1^, while the two benzenediols, methoxyhydroquinone (73.7) and 3-methylcatechol (73.9), represent the reactive lower boundary. The most significant breach of basic substituent intuition is 2,4-di-tert-butylphenol: devoid of an oxygenated substituent, it was anticipated to be close to the phenol baseline, yet it dissociates at 77.1 kcal mol^–1^ due to the stabilizing effect of the two adjacent tert-butyl groups on the phenoxyl radical through hyperconjugation, highlighting that radical stabilization, rather than ground-state electronics, dictates the bond dissociation energy (BDE).

**Table 2:** First and Second O–H Bond Dissociation Enthalpies (kcal mol^–1^), Consensus Lipophilicity (log *P*), Hirshfeld Oxygen Spin Population of the Phenoxyl Radical, and Mechanistic Classification of the Nine Phenolics, Ordered by Ascending First O–H BDE^a^

compound	GC-MS origin	1st O–H BDE	2nd O–H BDE	log *P*	Hirshfeld O-spin	role
Methoxyhydroquinone	Areca SL18	73.7	61.0	0.74	0.278	Propagator
3-Methylcatechol	Areca SL17	73.9	69.2	1.43	0.294	Propagator
Syringol	Corncob SL16/Areca SL20	74.5		1.19	0.297	Terminator
Creosol	Corncob SL14	76.6		1.64	0.296	Terminator
4-Ethylguaiacol	Corncob SL15/Areca SL19	76.8		1.97	0.297	Terminator
2,4-Di-*tert*-butylphenol	Corncob SL18/Areca SL22	77.1		4.21	0.309	Terminator
Guaiacol	Corncob SL9/Areca SL12	77.3		1.31	0.302	Terminator
Guaiacylacetone	Areca SL23	77.9		1.30	0.295	Terminator
Phenol	Corncob SL3	83.2		1.45	0.352	Terminator

a GC-MS origin refers to the sample and serial-number entries of the corncob and *Areca catechu* composition tables (Tables S1 and S2).

### Sequential O–H Dissociation and the Quinone Cascade

The two benzenediols exhibit differences from the monophenols that are not reflected in the initial BDE ranking. The semiquinone intermediate contains an additional O–H bond that can be cleaved to yield a stable quinone following the primary hydrogen transfer. In the case of methoxyhydroquinone, the second bond dissociation energy decreases to 61.0 kcal mol^–1^ (12.7 kcal mol^–1^ lower than the first), while for 3-methylcatechol, it is 69.2 kcal mol^–1^, both significantly lower than the sole bond dissociation energy of any monophenol. The progressive decrease in the second bond dissociation energy indicates the thermodynamic stability of the resultant quinone and demonstrates that these catechol/hydroquinone-like phenolics can sequentially donate two hydrogen atoms, with the second transfer being more advantageous than the first, a characteristic of the most potent phenolic antioxidants and a recognized trait of catechols as sequential hydrogen donors^6,32^. Based on this, the classification bifurcates into two operational categories: monophenols perform as radical terminators (donating one hydrogen atom to a stabilized phenoxyl radical), whereas benzenediols serve as propagators capable of redox cycling among diol, semiquinone, and quinone states (Table 2).

### Substituent Effects and Hammett Analysis

To establish the reactivity axis on a quantitative and transferable basis and to evaluate if the calculated O–H bond dissociation energies adhere to traditional physical-organic principles, the dataset was expanded from nine natural components to twenty-one phenols by incorporating 12 mono-substituted derivatives that cover a broad electronic spectrum. All modifications were handled in the same manner as the initial geometry optimization and analytical frequency assessment at the B3LYP-D3BJ/def2-TZVP/CPCM­(water) level, employing an open-shell (UKS) approach for the phenoxyl radicals, ensuring the expanded dataset is internally coherent. The augmentations include **eight** (it should be seven) para-substituted phenols (4-NH_2_, 4-OH, 4-OMe, 4-tBu, 4-Cl, 4-Br, 4-NO_2_), three meta-substituted phenols (3-Me, 3-Cl, 3-NO_2_), and two ortho-substituted phenols (2-Me, 2-Cl). Within the expanded dataset, the initial O–H bond dissociation energy varies from 70.6 kcal mol^–1^ (4-aminophenol) to 89.5 kcal mol^–1^ (4-nitrophenol), including a range of approximately 19 kcal mol^–1^ that aligns systematically with the electronic properties of the substituents.

Graphing the determined O–H bond dissociation energy (BDE) against the Hammett constant σ^+^ (Figure 1) reveals a remarkable linear relationship for the para series (BDE = 8.3σ^+^ + 82.5, *r* = 0.987), with a reaction constant ρ approximately +8.3 kcal mol^–1^ for each σ unit. The positive ρ signifies that electron-withdrawing groups increase the O–H bond dissociation energy, destabilizing the phenoxyl radical and hindering hydrogen atom transfer, while electron-donating groups decrease it, stabilizing the radical and promoting HAT reactivity, which aligns with the anticipated behavior of a HAT-driven radical-scavenging mechanism. The association with σ^+^ instead of σ indicates through-resonance stabilization of the phenoxyl radical by para π-donor substituents. The meta series follows the same trend concerning σ_m, whereas ortho-substituted phenols are presented separately due to the steric and intramolecular hydrogen-bonding influences introduced by ortho substituents (such as the O–H···Cl interaction in 2-chlorophenol), which are not reflected in the σ/σ^+^ constants obtained from meta/para data, thus, these instances diverge from the para/meta correlation. Significantly, the expansion of the data set from nine to twenty-one compounds considerably enhances the statistical foundation of the structure-reactivity correlations shown here, enabling a quantitative interpretation of the substituent effects instead of a qualitative one.

**Figure 1:**
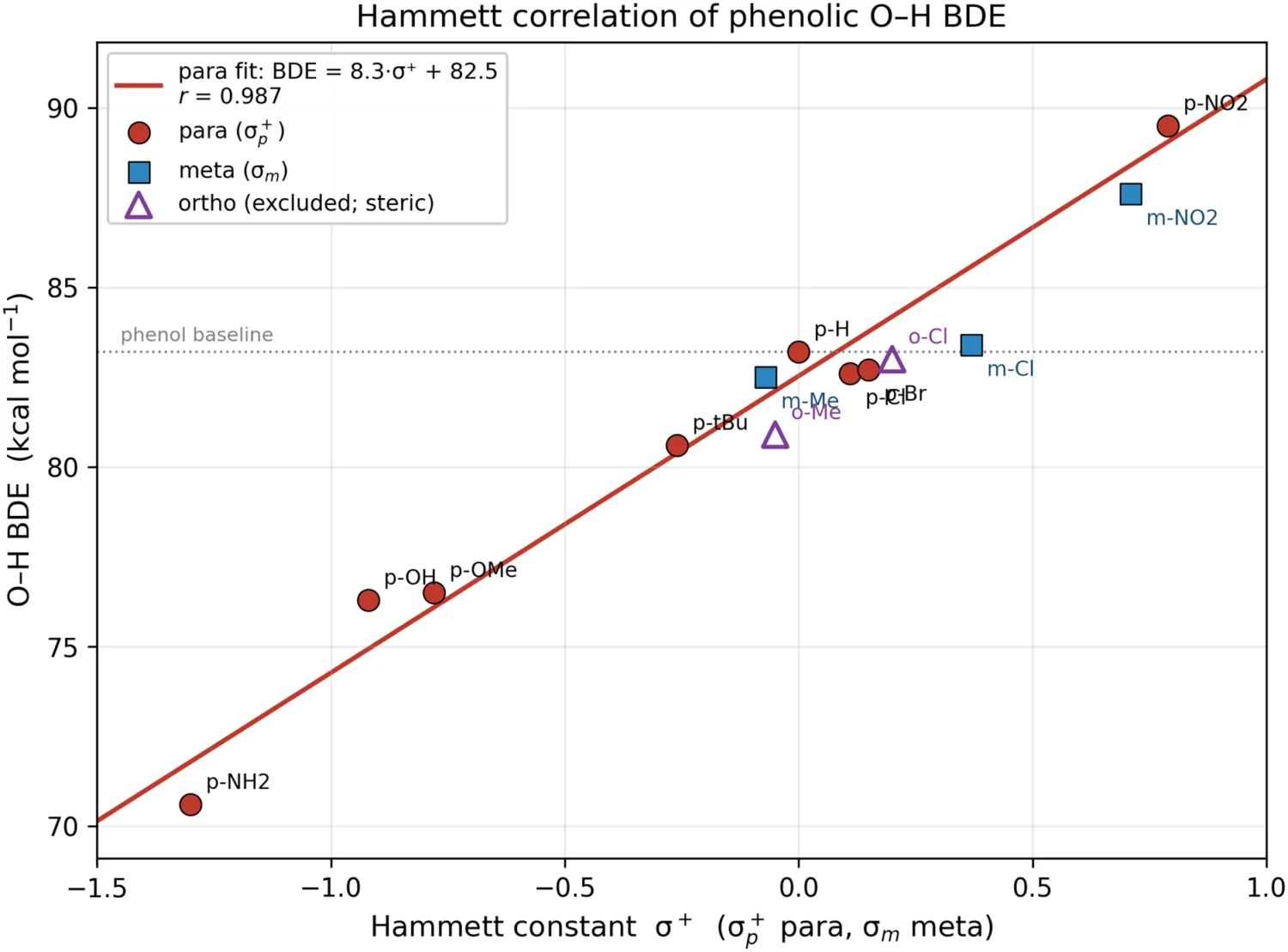
Hammett correlation of the calculated phenolic O–H BDE with σ^+^ (para, filled circles) and σ_m (meta, squares). The solid line is the para-series least-squares fit (BDE = 8.3σ^+^ + 82.5, *r* = 0.987, ρ ≈ +8.3 kcal mol^–1^). Ortho-substituted phenols (open triangles) are shown for completeness but excluded from the regression owing to steric and intramolecular hydrogen-bonding effects. The dotted line marks the phenol baseline.

### Benchmarking against Experimental BDEs

To evaluate the dependability of the methodology, the computed O–H bond dissociation energies were juxtaposed with empirical literature values for the group of compounds with trustworthy measurements. The gas-phase O–H bond dissociation energy (BDE) of phenol has been rigorously assessed at 86.7 ± 0.7 kcal mol^–1^_,_ ^35^ while the current measurement of 83.2 kcal mol^–1^ is approximately 3.5 kcal mol^–1^ lower. A similar systematic underestimation (≈4–5 kcal mol^–1^) is extensively recorded for B3LYP-class functionals, indicating a recognized propensity to excessively stabilize the closed-shell parent in comparison to the open-shell phenoxyl radical, this does not influence relative bond strengths, as these errors predominantly offset each other. In a comparative context (ΔBDE in relation to phenol), the alignment with photoacoustic-calorimetry data^36^ is satisfactory: guaiacol and 4-methoxyphenol are calculated to be 5.9 and 6.7 kcal mol^–1^ lower than phenol, compared to experimental measurements of 4.0 and 4.9 kcal mol^–1^, accurately reflecting both the trend and sequence of the substituent influence. The structure-reactivity analysis established in this study depends on the relative ranking of O–H BDEs instead of their absolute values, so this degree of concordance endorses the protocol’s application for its intended comparative function. Absolute bond dissociation energies (BDEs) are thereby presented as internally coherent relative indicators instead of forecasts of empirical bond strengths, and the related assertions in the abstract and conclusions have been articulated in this manner.

### Spin-Density Delocalization

To establish a physical foundation for the BDE sequence, the distribution of unpaired electrons in each phenoxyl radical was analyzed using its spin-density wavefunction. Atomic spin distributions derived from Hirshfeld, Löwdin, and Mulliken methodologies totaled ≈1.0 in every instance, validating a pure doublet. The unpaired spin on the phenolic oxygen adheres closely to the BDE hierarchy (Figure 2a): phenol, the least reactive compound, exhibits the most localized oxygen radical (Hirshfeld O-spin 0.352), whereas methoxyhydroquinone, the most reactive, displays the most delocalized spin (0.278). The Hirshfeld oxygen spin across the nine compounds exhibits a correlation with the initial O–H bond dissociation energy, yielding a Pearson r of 0.911, which is more robust and reliable than the Löwdin (0.842) or Mulliken (0.856) metrics. Consequently, the degree of spin localization on the phenoxyl oxygen serves as a tangible predictor of O–H bond strength, where methoxy and hydroxyl substitutions diminish the bond dissociation energy due to enhanced conjugation facilitating spin delocalization. Spin-density isosurfaces for all radicals are included in the Supporting Information (Figure S1).

**Figure 2:**
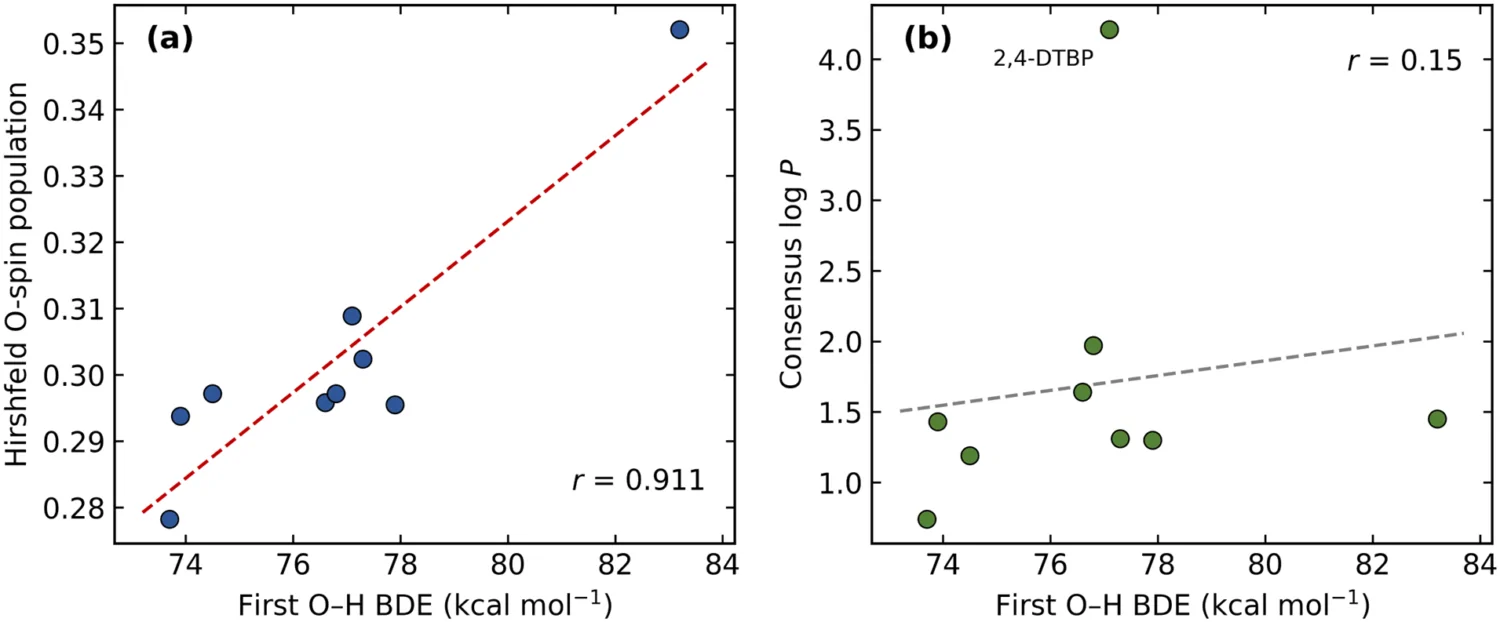
(a) Hirshfeld oxygen spin population of the phenoxyl radical versus the first O–H bond dissociation enthalpy (Pearson *r* = 0.911), the dashed line is a linear fit. (b) Consensus log *P* versus the first O–H BDE (*r* = 0.15), showing that lipophilicity and radical reactivity are orthogonal structure-activity axes.

### Lipophilicity

The agreed-upon octanol-water partition coefficients span from the extremely hydrophilic methoxyhydroquinone (0.74) to the significantly lipophilic 2,4-di-*tert*-butylphenol (4.21), while the other substances fall within the 1.2-2.0 range (Table 2). Significantly, log *P* and the initial O–H bond dissociation energy are statistically independent (Pearson *r* = 0.15, Figure 2b), indicating that membrane affinity and radical reactivity are orthogonal characteristics. In the current compilation, the optimal pairing of low BDE and high log *P* remains unrepresented: the most potent hydrogen-atom donors are concurrently the most hydrophilic, whereas the most lipophilic substance is merely a moderate donor.

### Molecular Docking against CYP51

To investigate a particular molecular target, the nine phenolic compounds were positioned within the active region of *C. albicans* CYP51 (PDB 5TZ1, 2.00 Å). The re-docking of the co-crystallized inhibitor VT-1161 replicated the crystallographic binding conformation (heavy-atom RMSD < 2 Å, Figure S2), confirming the grid definition and scoring function, and yielded the highest affinity among all ligands, −12.2 kcal mol^–1^. To contextualize the phenolic binding energies in a clinically relevant manner, the primary azole antifungal fluconazole was docked under the same conditions as a positive control, exhibiting a binding energy of −8.4 kcal mol^–1^, as fluconazole is the least potent CYP51 inhibitor among clinically utilized azoles, it serves as a conservative lower reference for active-site binding. The anticipated affinities for the nine phenolic compounds varied from −5.0 kcal mol^–1^ (phenol) to −7.6 kcal mol^–1^ (2,4-di-*tert*-butylphenol) (Table 3, and Figure 3a), all of which were inferior to the controls. Significantly, none of the phenolic compounds include the heme-coordinating azole nitrogen characteristic of genuine CYP51 inhibitors, hence, their affinity aligns with nonspecific lipophilic interactions rather than mechanism-based inhibition. The hierarchy is determined by molecular dimensions and lipophilicity rather than radical reactivity: binding affinity is associated with consensus log *P* (Pearson *r* = −0.76, *p* = 0.017, Figure 3b) but not with the O–H bond dissociation energy (*r* = 0.19, *p* = 0.62) or the Hirshfeld oxygen spin (*r* = 0.28, *p* = 0.46). Docking thus evaluates the identical lipophilicity axis previously delineated and is perpendicular to the radical-reactivity axis.

**Figure 3:**
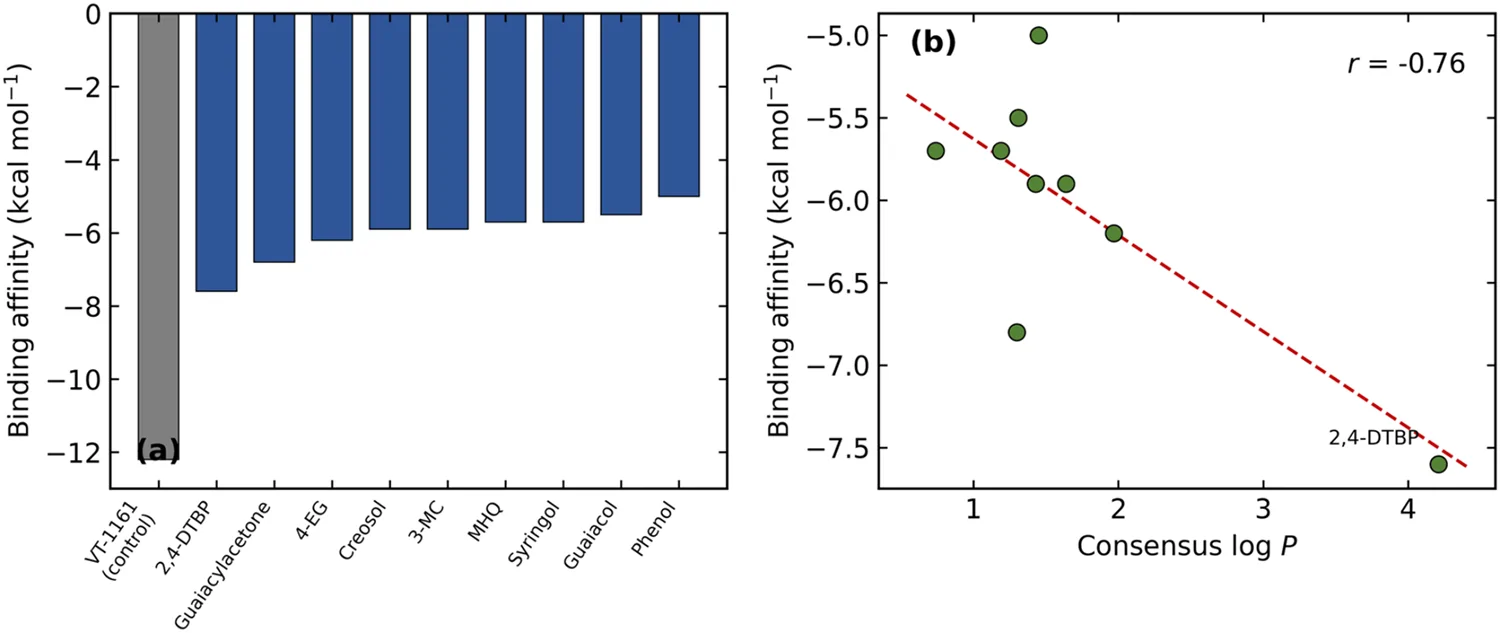
(a) AutoDock Vina binding affinities of the nine phenolics against *C. albicans* CYP51, ranked strongest to weakest, with the re-docked VT-1161 inhibitor shown as a control. (b) Binding affinity versus consensus log *P* (Pearson *r* = −0.76), the dashed line is a linear fit. Affinity tracks lipophilicity but is independent of the O–H BDE and radical spin density.

**Table 3:** Top-Ranked AutoDock Vina Binding Affinities against *C. albicans* CYP51 (PDB 5TZ1), with the Re-Docked Native Inhibitor VT-1161 (Protocol Validation) and the Clinical Azole Fluconazole (Positive Control)^a^

compound	binding affinity (kcal mol^–1^)	log *P*	role
VT-1161 (oteseconazole)	–12.2		Native co-crystal inhibitor (protocol validation)
Fluconazole	–8.4		First-line clinical azole (positive control)
2,4-Di-*tert*-butylphenol	–7.6	4.21	phenolic
Guaiacylacetone	–6.8	1.30	phenolic
4-Ethylguaiacol	–6.2	1.97	phenolic
Creosol	–5.9	1.64	phenolic
3-Methylcatechol	–5.9	1.43	phenolic
Syringol	–5.7	1.19	phenolic
Methoxyhydroquinone	–5.7	0.74	phenolic
Guaiacol	–5.5	1.31	phenolic
Phenol	–5.0	1.45	phenolic (parent)

a All ligands were docked with identical grid (center 64.24, 67.86, 2.74 Å, box 25.95 × 25.59 × 25.46 Å^3^) and exhaustiveness (16), each value is the top-ranked (most negative) of nine generated poses.

### Two Independent Modes of Action

The chemicals analyzed in this study may, theoretically, enhance antifungal activity via two unique molecular pathways, which we address individually. The initial aspect is radical chemistry: the transfer of a hydrogen atom from the phenolic O–H, measured by the O–H bond dissociation energy and the resultant spin delocalization of the phenoxyl radical. The second mechanism involves direct enzyme inhibition: interaction within the CYP51 active site, evaluated through docking and mostly influenced by lipophilicity and steric compatibility rather than O–H bond strength. These pathways are chemically distinct, and we assert no correlation between a compound’s ability to donate a hydrogen atom and its affinity for CYP51. The independence is numerical: throughout the series, the reactivity descriptor (O–H BDE) and the lipophilicity descriptor (consensus log *P*) exhibit minimal correlation (Pearson *r* = 0.15), indicating that the two axes are statistically orthogonal, and a compound’s location on one axis provides no insight into its location on the other. This orthogonality constitutes a fundamental finding of the study instead of a presumption, and it accounts for the presentation of the two mechanisms in distinct parts. When compared to the azole controls, the phenolic docking scores are moderate and, in the absence of the heme-coordinating azole structure, suggest that direct CYP51 suppression is likely a secondary factor, with radical/membrane chemistry being the more probable primary mechanism.

### The Propagator/Terminator Framework: Thermodynamic Definition

In a radical-chain autoxidation, the chain is sustained by peroxyl radicals (ROO^•^), which propagate through the abstraction of hydrogen from the substrate (ROO^•^ + RH → ROOH + R^•^). A phenol participates by transferring its O–H hydrogen to the peroxyl radical (ArOH + ROO^•^ → ArO^•^ + ROOH), the determination of whether this disrupts or just propagates the chain is influenced by the O–H bond dissociation energy of the phenol in comparison to that of the peroxyl radical, which serves as the inherent benchmark for the reaction. Consequently, we delineate the two operational categories based on a singular thermodynamic standard rooted in the peroxyl reference bond. The O–H bond dissociation energy (BDE) for *tert*-butyl hydroperoxide (*t*-BuOO-H) was calculated at the same theoretical level (79.9 kcal mol^–1^), it is crucial to compute the reference in the same manner as the phenols due to a systematic offset that leads to an underestimation of absolute O–H BDEs, necessitating that the peroxyl reference aligns with the same scale. The driving force ΔBDE is defined as BDE­(ArO-H) – BDE­(ROO-H), a phenol acts as a terminator when ΔBDE < 0 (the exothermic H atom transfer sequesters the chain carrier, and the resultant phenoxyl radical is sufficiently stable to prevent re-abstracting from the substrate) and functions as a propagator when ΔBDE ≥ 0. When applied to the dataset, this criterion demonstrates chemical consistency: all natural wood-vinegar phenolics are below the threshold as terminators, with ΔBDE ranging from −2.0 kcal mol^–1^ (guaiacylacetone) to −6.2 kcal mol^–1^ (methoxyhydroquinone), while benzenediols serve as the most potent H atom donors, among the substituent probes, robust π-donors further broaden the range (4-aminophenol, −9.3 kcal mol^–1^), the unsubstituted parent phenol (+3.3 kcal mol^–1^) is positioned just above the limit, aligning with its established weak chain-breaking capability, and the electron-deficient nitrophenols clearly act as propagators (3-nitrophenol +7.7, 4-nitrophenol +9.6 kcal mol^–1^). The secondary axis, spin delocalization, enhances the categorization within the terminator class: for a specific driving force, a more broadly delocalized phenoxyl radical (reduced oxygen spin population) exhibits greater stability and serves as a more effective terminator. In rigorous radical-chain kinetics, the terms “propagation” and “termination” refer to fundamental steps, the terminology employed here serves as functional descriptors of phenol activity, with “terminator” indicating a chain-breaking (inhibitory) antioxidant and “propagator” representing a phenol that does not disrupt the chain and whose radical can re-engage in propagation, aligning seamlessly with the chain-breaking-antioxidant model established by Ingold, Pedulli, Valgimigli, and their associates.^35^

### Competition between HAT, SET-PT, and SPLET Pathways in Aqueous Media

Phenolic hydrogen donation can occur via three thermodynamically interrelated routes that yield a shared phenoxyl-radical product: direct hydrogen atom transfer (HAT, determined by the O–H bond dissociation energy), single-electron transfer followed by proton transfer (SET-PT, influenced by the ionization potential), and sequential proton-loss electron transfer (SPLET, dictated by the enthalpies of proton dissociation and electron transfer).^32^ In nonpolar settings, HAT generally prevails, while in polar, ionizing solvents like water, the SPLET and SET-PT pathways may compete due to the solvent’s stabilization of charged intermediates,^39^ thus, the aqueous continuum model employed here does not solely affirm HAT as the predominant mechanism, nor do we assert that it is. The current examination centers on the HAT mechanism for three primary reasons: the Propagator/Terminator framework pertains to the interruption of radical chains via hydrogen-atom transfer, which is fundamentally HAT, the biologically significant site for these lipophilic phenolics is the low-polarity fungal membrane, where HAT is anticipated to prevail over charge-separated pathways, aligning with the distinct role of lipophilicity identified herein, and, crucially for a comparative analysis, the O–H bond dissociation energy (BDE) remains a pertinent reactivity indicator regardless of the pathway utilized, as all three pathways yield the same phenoxyl radical and encompass the radical-stabilization aspect quantified by the BDE, thereby maintaining the relative ranking. A comprehensive mechanistic analysis in bulk water would also necessitate the ionization potential (SET-PT) and the enthalpies of proton dissociation and electron transfer (SPLET), which extend beyond the current focus on bond dissociation energies and spin, indicating a logical avenue for future research.

## Discussion

The examination delineates the antifungal phenolic fraction into a coherent structure-reactivity framework centered on two distinct characteristics. The initial factor is the simplicity of hydrogen atom donation, assessed through the O–H bond dissociation energy and fundamentally linked to the dispersion of the phenoxyl radical’s unpaired electron from oxygen, the robust correlation between spin-BDE (*r* = 0.911) indicates that these represent two perspectives of the identical electronic phenomenon, while the Hammett analysis (ρ ≈ +8.3, *r* = 0.987) establishes this axis on a quantitative substituent basis. The second factor is lipophilicity, which regulates membrane penetration and is statistically independent of the first (*r* = 0.15). In this dual-axis framework, the components are categorized into monophenolic radical terminators and benzenediol propagators, whose diminished second O–H bond dissociation energies facilitate a quinone redox cascade that generates rather than just neutralizes reactive species. This model produces a definitive, verifiable forecast: a formulation enriched in propagator-class benzenediols is anticipated to exhibit greater antifungal efficacy than one comprising solely monophenolic terminators.

The Areca catechu vinegar comprised two propagator-class benzenediols, namely 3-methylcatechol and methoxyhydroquinone, which were not present in the corncob profile (which had just monophenolic terminators). The highly lipophilic compound 2,4-di-*tert*-butylphenol is found in both samples, thereby failing to differentiate the two profiles. The substances distinguishing the two vinegars are specifically the propagators recognized here as mechanistically unusual, suggesting that their pro-oxidant redox cycling would enhance the antifungal efficacy of the benzenediol-containing formulation. This forecast is derived from the calculated descriptors and has not been evaluated in this context, as the extracts consist of intricate combinations and the antifungal activities at the chemical level were not assessed. Collectively, the descriptors imply a multi-target mechanism of action: lipophilic components infiltrate and disrupt the fungal membrane, low-BDE monophenols neutralize membrane lipid-peroxidation radicals through hydrogen atom transfer, and the benzenediol derivatives can elicit oxidative stress through semiquinone/quinone cycling in the iron-abundant, aerobic environment of the fungal cell. This convergent behavior is typical of natural phenolic antifungals and partially elucidates the consistent efficacy of phenolic-rich extracts, even though the individual components possess moderate potency.^4,8^ It further justifies the prevalent application of the O–H bond dissociation energy (BDE) as the primary indicator of phenolic radical activity,^6,7^ while suggesting that BDE alone is inadequate for benzenediols, where the second dissociation is the crucial mechanistic step.

Numerous prior investigations into the antifungal properties of wood-vinegar, encompassing those on palm, litchi, and Cinnamomum vinegars,^11–14^ delineate composition using GC-MS and evaluate the unrefined liquid, ascribing efficacy to the phenolic component. The current study enhances this by addressing that proportion at the electronic-structure level and designating a specific mechanistic role to each component. Numerous constraints hinder these results. The descriptor set exhibits internal consistency, however, the natural set is limited to nine compounds. While the expanded Hammett series of 21-compounds enhances the analysis of substituents, antifungal potencies at the compound level were not assessed, rendering the reported relationships as structure-property correlations instead of confirmed quantitative structure-activity relationships, and the correlations derived from the nine-compound core (including the *r* = 0.911 spin-BDE relationship) should be interpreted with due caution. The global reactivity descriptors exhibit significant intercorrelation and collectively provide a singular effective electronic dimension. The CPCM­(water) continuum simulates a watery environment while disregarding the anisotropic, low-dielectric core of a lipid membrane. Ultimately, the differentiation between propagators and terminators is based on gas and solution-phase thermodynamics, and the genuine generation of reactive oxygen species within fungal cells has yet to be validated. These limitations delineate the subsequent stages. The most straightforward approach involves conducting compound-level antifungal bioassays against pertinent fungal infections, which would transform the current structure-property relationships into a cross-validated QSAR and empirically evaluate the hypothesis that the Areca-specific benzenediol propagators exhibit disproportionately high activity. The docking outcomes suggest that the direct inhibition of CYP51, the established azole target,^3^ serves primarily as a secondary mechanism for these phenolic compounds: their binding affinities (−5.0 to −7.6 kcal mol^–1^) are inferior to that of the clinical azole fluconazole (−8.4 kcal mol^–1^) and the native inhibitor VT-1161 (−12.2 kcal mol^–1^), are influenced more by lipophilicity than by radical reactivity, and do not possess the heme-coordinating azole structure, indicating that radical-mediated membrane chemistry is likely the predominant mode of action. From a design perspective, the vacant low-BDE/high-log *P* area of the structure-activity map offers a concrete optimization objective.

## Conclusions

The antifungal chemistry of nine phenolic compounds from corncob and Areca catechu wood vinegars was examined at the molecular level using DFT, spin-density analysis, lipophilicity prediction, and molecular docking, while the reactivity spectrum was broadened to include twenty-one phenols to formulate a quantitative Hammett relationship. The initial O–H bond dissociation energy (BDE) ranges from 73.7 to 83.2 kcal mol^–1^ for the natural dataset (70.6 to 89.5 in the expanded series), with benzenediols exhibiting the highest reactivity and phenol serving as the reference point, the para-series O–H BDE shows a correlation with σ^+^ (ρ ≈ +8.3, *r* = 0.987), and computed values align with experimental relative BDEs once a systematic B3LYP deviation is considered. The Hirshfeld oxygen spin population of the phenoxyl radical correlates with the bond dissociation energy (*r* = 0.911), indicating that spin delocalization is the underlying cause of this trend, whereas lipophilicity shows no correlation with BDE (*r* = 0.15), resulting in two orthogonal design dimensions. The benzenediols exhibit diminished second O–H bond dissociation energies, facilitating a quinone redox cascade, which allows for a classification, quantitatively compared to a peroxyl reference bond, into radical-terminating monophenols and redox-cycling propagators. This framework forecasts a compositional foundation for the disparity in antifungal efficacy between the two vinegars, with the two propagators present solely in the Areca catechu profile and lacking in corncob, a hypothesis readily verifiable through compound-level assays, and designates the unutilized low-BDE/high-log *P* area as a target for optimization. Docking reveals that the phenolics have limited binding affinity to CYP51, driven by lipophilicity (−5.0 to −7.6 kcal mol^–1^), which is inferior to fluconazole (−8.4) and VT-1161 (−12.2 kcal mol^–1^), suggesting that direct enzyme inhibition is at best a secondary mechanism, with radical-mediated reactions prevailing. Compound-level bioassays represent the crucial subsequent phase in developing a predictive, target-oriented model.

## Materials and Methods

### Sample Collection and Wood-Vinegar Preparation

Zea mays corncobs and Areca catechu stems (Khulna, Bangladesh) were gathered, cut into diminutive fragments, and air-dried under sunlight for a duration of 15 days. Wood vinegar was obtained through the pyrolysis of desiccated biomass in a round-bottom flask, which was heated using a heating mantle linked to a water-cooled condenser, the vapor produced was condensed into a brown liquid known as wood vinegar. Heating persisted until the emergence of char and black tar occurred.^10^ The two condensates were stored independently in sealed containers and examined after a period of 45 days.

### GC-MS/MS Analysis

The composition was assessed using a gas chromatograph-tandem mass spectrometer (Shimadzu TQ8040) equipped with a Rxi-5 ms column (30 m × 0.25 mm i.d., 0.25 μm film), with the oven designed to ramp from 50 to 300 °C utilizing helium as the carrier gas. Constituents were recognized by referencing the NIST/EPA/NIH Mass Spectral Library. Comprehensive inventories are shown in Tables S1 and S2.

### Selection of Phenolic Compounds

The in silico assessment was conducted on nine phenolic compounds from three distinct structural categories, chosen from the 25 components identified in each wood vinegar: phenol, 2-methoxyphenol (guaiacol), 2-methoxy-4-methylphenol (creosol), 4-ethyl-2-methoxyphenol (4-ethylguaiacol), 2,6-dimethoxyphenol (syringol), 1-(4-hydroxy-3-methoxyphenyl)­propan-2-one (guaiacylacetone), 2,4-di-*tert*-butylphenol, 3-methylbenzene-1,2-diol (3-methylcatechol), and 2-methoxybenzene-1,4-diol (methoxyhydroquinone). The term “Creosol” is employed accurately in its intended meaning (2-methoxy-4-methylphenol, or 4-methylguaiacol) and is not an alternative spelling of cresol. The Hammett analysis was expanded to include 12 substituted phenols: para substitutions of 4-amino-, 4-hydroxy-, 4-methoxy-, 4-*tert*-butyl-, 4-chloro-, 4-bromo-, and 4-nitrophenol, meta substitutions of 3-methyl-, 3-chloro-, and 3-nitrophenol, and ortho substitutions of 2-methyl- and 2-chlorophenol. The chemical configurations of all examined compounds are illustrated in Figure 4. Common nomenclature will be employed forward.

**Figure 4:**
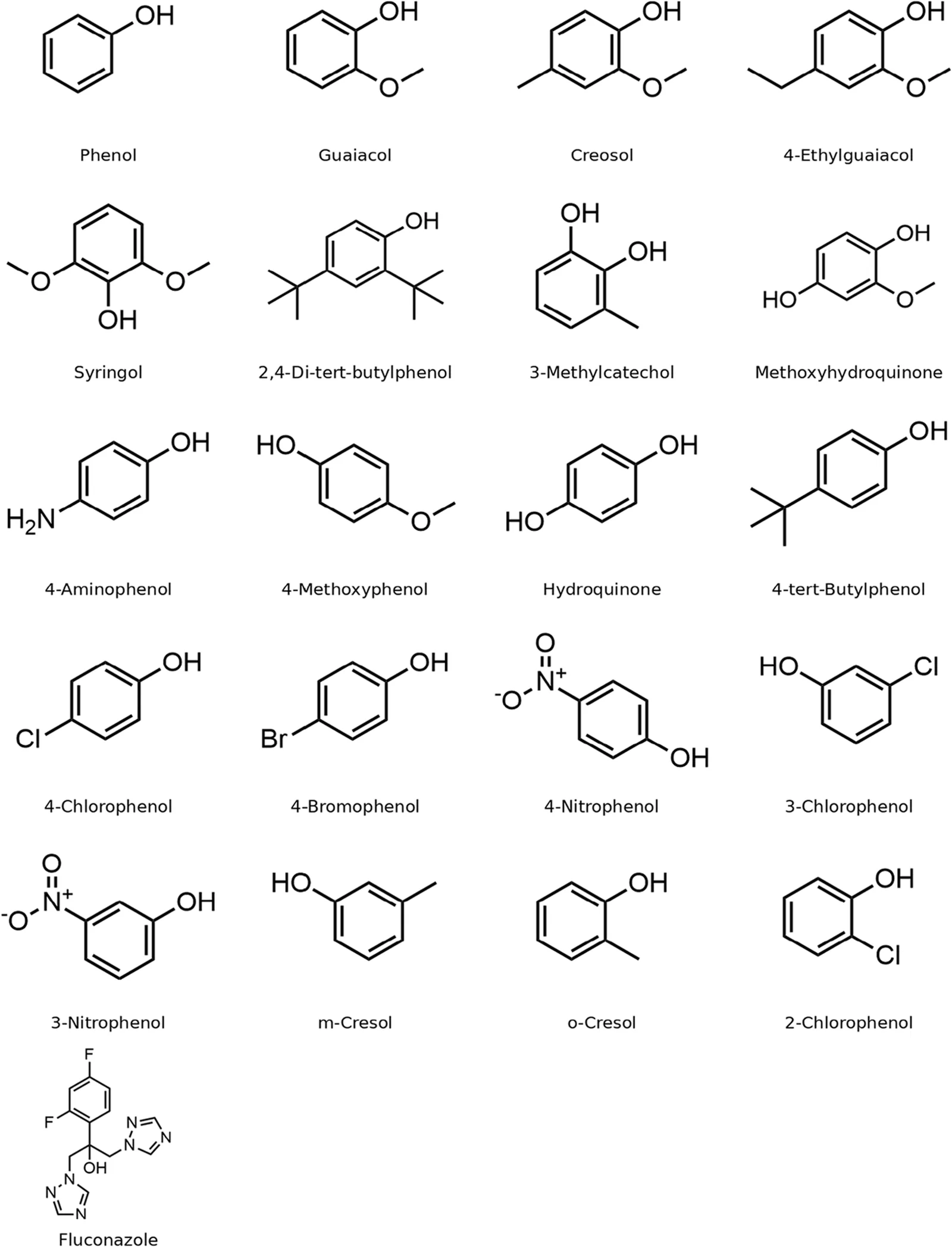
Structures of the studied compounds: the nine wood-vinegar phenolics (top rows), the 12 substituted phenols added for the Hammett analysis, and the clinical azole fluconazole used as the docking positive control.

### Density Functional Theory Calculations

The preliminary geometry were constructed and pre-optimized utilizing the MMFF94 force field in Avogadro 2.0.0.^18^ All quantum-chemical calculations employed ORCA 6.1.1 ^19^ on a Windows 11 computer (Intel Core i7-1255U, 16 GB RAM). Geometry optimizations and analytical frequency computations were performed at the B3LYP level,^20,21^ incorporating Grimme’s D3 dispersion and Becke-Johnson damping (D3BJ),^22^ utilizing the def2-TZVP basis set,^23^ and employing RIJCOSX with the def2/J auxiliary basis, aqueous solvation was modeled using CPCM­(water).^24^ The methodologies TightSCF, TightOpt, and DEFGrId3 were utilized. Frequency assessments were conducted using the same theoretical framework as the optimizations, confirming that all optimized parent compounds and phenoxyl radicals represented genuine minima devoid of imaginary frequencies, a limited number of structures that initially exhibited low-frequency imaginary modes related to methyl or nitro torsion were re-optimized by rotating the problematic group by 30–40° and/or employing a more refined grid until all frequencies became real. The 12 substituted phenols in the Hammett series were calculated in the same manner. The absolute BDEs derived from this approach were compared to rigorously assessed experimental values^35,36^ and are presented as internally coherent relative descriptors (see to Benchmarking).^38^

### Global Reactivity Descriptors

Descriptors were computed from frontier-orbital energies in Koopmans approximation.^25^ With IP = −*E*(HOMO) and EA = −*E*(LUMO): Δ*E* = *E*(LUMO) – *E*(HOMO), χ = (IP + EA)/2 and μ = −χ, η = (IP – EA)/2 and *S* = 1/η, ω = μ^2^/(2η).

### O–H Bond Dissociation Enthalpies

The homolytic O–H bond dissociation energy (BDE) was calculated for the reaction ArO-H → ArO^•^ + H^•^ as BDE = H­(ArO^•^) + H­(H^•^) – H­(ArOH), utilizing enthalpic values at 298.15 K derived from thermochemical evaluation. Phenoxyl radicals were produced by removing the phenolic hydrogen and subsequently re-optimized as open-shell doublets (unrestricted Kohn-Sham, charge 0, multiplicity 2) with frequency analysis, ⟨*S* ^2^⟩ was ≈0.77 for each radical. For the benzenediols, both hydroxyl positions were evaluated, and the lesser value was considered effective, the second O–H bond dissociation energy (from semiquinone to quinone) was calculated in a similar manner. For the classification of propagators and terminators, the O–H bond dissociation energy (BDE) of *tert*-butyl hydroperoxide (*t*-BuOO-H) was calculated in the same manner as a peroxyl reference (79.9 kcal mol^–1^), with the driving force for classification defined as ΔBDE = BDE­(ArO-H) – BDE­(ROO-H).

### Hammett Analysis

Computed initial O–H bond dissociation energies were correlated with Hammett substituent constants (σ^+^ for para, σ_m for meta) derived from the established compilation.^37^ The reaction constant ρ and the correlation coefficient were derived via a least-squares analysis of the para series, ortho-substituted phenols were graphed but omitted from the regression due to steric and intramolecular hydrogen-bonding influences.

### Spin-Density Analysis

The distributions of unpaired electrons were examined using Multiwfn.^26^ The ORCA wavefunction (.gbw) was transformed into Molden format (orca_2mkl), and the spin density was assessed on a grid; atomic spin populations were obtained using Hirshfeld partitioning and corroborated by Löwdin and Mulliken analyses, totaling around 1.0 per radical. UCSF ChimeraX^27^ produced isosurfaces at ±0.004 atomic units.

### Lipophilicity Prediction

The standard SMILES of each molecule were utilized to derive the consensus octanol-water partition coefficient (log *P*) from SwissADME,^15^ with the consensus representing the average of the individual prediction models.

### Molecular Docking

Docking was executed utilizing the *C. albicans* CYP51 catalytic domain co-crystallized with VT-1161 (PDB 5TZ1).^3^ The receptor was generated in UCSF ChimeraX^27^ by isolating a single protein chain, eliminating solvent, and preserving the heme prosthetic group along with its iron, subsequently transformed to PDBQT in PyRx^28^ with confirmation of heme/iron retention. Ligands were extracted from the DFT-optimized neutral structures and transformed into PDBQT format using the Open Babel interface **in** PyRx.^29^ Docking was conducted using AutoDock Vina,^30^ with a search box focused on the heme iron located at (*x*, *y*, *z*) = (64.24, 67.86, 2.74) Å, dimensions of 25.95 × 25.59 × 25.46 Å^3^, and an exhaustiveness setting of 16; uniform grid, box, and score settings were utilized for all ligands. Nine conformations were produced for each ligand, and the lowest affinity was documented. The co-crystallized VT-1161 was re-docked as a validation reference, while the clinical azole fluconazole was docked under the same conditions as a positive benchmark

### Statistical Analysis

The interrelations among descriptors were analyzed with Pearson and Spearman correlation coefficients, employing a correlation matrix to measure collinearity. Evaluations and graphical representations were conducted using Microsoft Excel and Python (NumPy/SciPy, Matplotlib).

## Supplementary Material



## Notes

The GC-MS analysis of both wood vinegars (Tables S1 and S2); spin-density isosurfaces of the calculated phenoxyl radicals (Figure S1); VT-1161 redocking validation with CYP51 (Figure S2); and for each optimized species (both parents and radicals), the concluding Cartesian coordinates, electronic (SCF) energies, zero-point and thermal corrections, and 298.15 K enthalpies, along with the count of imaginary frequencies and ⟨*S* ^2^⟩ for open-shell species (PDF)

## Abbreviations

BDE: bond dissociation enthalpy
CPCM: conductor-like polarizable continuum model
CYP51: sterol 14α-demethylase
D3BJ: Grimme D3 dispersion with Becke-Johnson damping
DFT: density functional theory
GC-MS: gas chromatography-mass spectrometry
HAT: hydrogen-atom transfer
HOMO: highest occupied molecular orbital
log *P*: octanol-water partition coefficient
LUMO: lowest unoccupied molecular orbital
QSAR: quantitative structure-activity relationship
ROS: reactive oxygen species
SET-PT: single-electron transfer-proton transfer
SMILES: simplified molecular-input line-entry system
SPLET: sequential proton-loss electron transfer
UKS: unrestricted Kohn-Sham

## References

[1] Fisher M. C., Hawkins N. J., Sanglard D., Gurr S. J. (2018). Worldwide Emergence
of Resistance to Antifungal Drugs Challenges Human Health and Food
Security. Science.

[2] Fisher M. C., Henk D. A., Briggs C. J., Brownstein J. S., Madoff L. C., McCraw S. L., Gurr S. J. (2012). Emerging
Fungal
Threats to Animal, Plant and Ecosystem Health. Nature.

[3] Hargrove T. Y., Friggeri L., Wawrzak Z., Qi A., Hoekstra W. J., Schotzinger R. J., York J. D., Guengerich F. P., Lepesheva G. I. (2017). Structural Analyses of *Candida albicans* Sterol 14α-Demethylase Complexed with Azole Drugs Address
the Molecular Basis of Azole-Mediated Inhibition of Fungal Sterol
Biosynthesis. J. Biol. Chem..

[4] Simonetti G., Brasili E., Pasqua G. (2020). Antifungal
Activity of Phenolic and
Polyphenolic Compounds from Different Matrices of *Vitis
vinifera*. Molecules.

[5] López M., Martínez F., Del Valle C., Ferrit M., Luque R. (2003). Study of Phenolic
Compounds as Natural Antioxidants by a Fluorescence Method. Talanta.

[6] Wright J. S., Johnson E. R., DiLabio G. A. (2001). Predicting
the Activity of Phenolic
Antioxidants: Theoretical Method, Analysis of Substituent Effects,
and Application to Major Families of Antioxidants. J. Am. Chem. Soc..

[7] Litwinienko G., Ingold K. U. (2007). Solvent Effects
on the Rates and Mechanisms of Reaction
of Phenols with Free Radicals. Acc. Chem. Res..

[8] Bortolomeazzi R., Sebastianutto N., Toniolo R., Pizzariello A. (2007). Comparative
Evaluation of the Antioxidant Capacity of Smoke Flavouring Phenols
by Crocin Bleaching Inhibition, DPPH Radical Scavenging and Oxidation
Potential. Food Chem..

[9] Sun Z., Fridrich B., de Santi A., Elangovan S., Barta K. (2018). Bright Side of Lignin Depolymerization:
Toward New Platform Chemicals. Chem. Rev..

[10] Wang S., Dai G., Yang H., Luo Z. (2017). Lignocellulosic Biomass Pyrolysis
Mechanism: A State-of-the-Art Review. Prog.
Energy Combust. Sci..

[11] Oramahi H. A., Yoshimura T., Diba F., Setyawati D., Nurhaida (2018). Antifungal and
Antitermitic Activities of Wood Vinegar from Oil Palm Trunk. J. Wood Sci..

[12] Kara M., Soylu S., Soylu E. M., Uysal A., Kurt Ş., Türkmen M. (2024). Determination of the Chemical Composition
and Antifungal Activity of Wood Vinegar (Pyroligneous Acid) against
the Onion Bulb Rot Disease Caused by *Fusarium proliferatum*. J. Crop Health.

[13] Yang J.-F., Yang C.-H., Liang M.-T., Gao Z.-J., Wu Y.-W., Chuang L.-Y. (2016). Chemical Composition,
Antioxidant, and Antibacterial
Activity of Wood Vinegar from *Litchi chinensis*. Molecules.

[14] Adfa M., Romayasa A., Kusnanda A. J., Avidlyandi A., Yudha S., Banon S., Gustian C. (2020). I. Chemical
Components,
Antitermite and Antifungal Activities of *Cinnamomum
parthenoxylon* Wood Vinegar. J. Korean Wood Sci. Technol..

[15] Daina A., Michielin O., Zoete V. (2017). SwissADME: A Free Web
Tool to Evaluate
Pharmacokinetics, Drug-Likeness and Medicinal Chemistry Friendliness
of Small Molecules. Sci. Rep..

[18] Hanwell M. D., Curtis D. E., Lonie D. C., Vandermeersch T., Zurek E., Hutchison G. R. (2012). Avogadro:
An Advanced Semantic Chemical
Editor, Visualization, and Analysis Platform. J. Cheminf..

[19] Neese F. (2022). Software Update:
The ORCA Program System, Version 5.0. WIREs
Comput. Mol. Sci..

[20] Becke A. D. (1993). Density-Functional
Thermochemistry. III. The Role of Exact Exchange. J. Chem. Phys..

[21] Lee C., Yang W., Parr R. G. (1988). Development
of the Colle-Salvetti
Correlation-Energy Formula into a Functional of the Electron Density. Phys. Rev. B.

[22] Grimme S., Ehrlich S., Goerigk L. (2011). Effect of the Damping Function in
Dispersion Corrected Density Functional Theory. J. Comput. Chem..

[23] Weigend F., Ahlrichs R. (2005). Balanced Basis Sets
of Split Valence, Triple Zeta Valence
and Quadruple Zeta Valence Quality for H to Rn: Design and Assessment
of Accuracy. Phys. Chem. Chem. Phys..

[24] Barone V., Cossi M. (1998). Quantum Calculation
of Molecular Energies and Energy Gradients in
Solution by a Conductor Solvent Model. J. Phys.
Chem. A.

[25] Parr R. G., Szentpály Lv., Liu S. (1999). Electrophilicity Index. J. Am. Chem. Soc..

[26] Lu T., Chen F. (2012). Multiwfn: A Multifunctional
Wavefunction Analyzer. J. Comput. Chem..

[27] Pettersen E. F., Goddard T. D., Huang C. C., Meng E. C., Couch G. S., Croll T. I., Morris J. H., Ferrin T. E. (2021). UCSF ChimeraX: Structure
Visualization for Researchers, Educators, and Developers. Protein Sci..

[28] Dallakyan, S. ; Olson, A. J. Small-Molecule Library Screening by Docking with PyRx. In Chemical Biology: Methods and Protocols, Methods in Molecular Biology; Humana Press: New York, 2015; Vol. 1263, pp 243–250 10.1007/978-1-4939-2269-7_19. 25618350

[29] O’Boyle N. M., Banck M., James C. A., Morley C., Vandermeersch T., Hutchison G. R. (2011). Open Babel: An Open Chemical Toolbox. J. Cheminf..

[30] Trott O., Olson A. J. (2010). AutoDock Vina: Improving the Speed and Accuracy of
Docking with a New Scoring Function, Efficient Optimization, and Multithreading. J. Comput. Chem..

[31] Nenadis N., Tsimidou M. Z. (2012). Contribution of
DFT Computed Molecular Descriptors
in the Study of Radical Scavenging Activity Trend of Natural Hydroxybenzaldehydes
and Corresponding Acids. Food Res. Int..

[32] Amić A., Mastiľák Cagardová D. (2022). DFT Study of the Direct Radical Scavenging
Potency of Two Natural Catecholic Compounds. Int. J. Mol. Sci..

[33] Kalinoski R. M., Shao Q., Shi J. (2024). Predicting Antimicrobial Properties
of Lignin Derivatives through a Combined Data-Driven and Experimental
Approach. Front. Ind. Microbiol..

[34] Jadhav A. K., Khan P. K., Karuppayil S. M. (2020). Phytochemicals
as Potential Inhibitors
of Lanosterol 14α-Demethylase (CYP51) Enzyme: An In Silico Study
on Sixty Molecules. Int. J. Appl. Pharm..

[35] Mulder P., Korth H.-G., Pratt D. A., DiLabio G. A., Valgimigli L., Pedulli G. F., Ingold K. U. (2005). Critical
Re-evaluation of the O-H
Bond Dissociation Enthalpy in Phenol. J. Phys.
Chem. A.

[36] de
Heer M. I., Korth H.-G., Mulder P. (1999). Poly Methoxy Phenols
in Solution: O-H Bond Dissociation Enthalpies, Structures, and Hydrogen
Bonding. J. Org. Chem..

[37] Hansch C., Leo A., Taft R. W. (1991). A Survey
of Hammett Substituent Constants and Resonance
and Field Parameters. Chem. Rev..

[38] Blanksby S. J., Ellison G. B. (2003). Bond Dissociation
Energies of Organic Molecules. Acc. Chem. Res..

[39] Litwinienko G., Ingold K. U. (2007). Solvent
Effects on the Rates and Mechanisms of Reaction
of Phenols with Free Radicals. Acc. Chem. Res..

