# Supplementary material for: In Silico Structure-Reactivity Analysis of Lignin-Derived Phenolics: O–H Bond Dissociation Enthalpies, Spin Delocalization, and a Propagator/Terminator Framework for Antifungal-Relevant Activity
Source: ACS Omega. 2026 Sep 8;11(37):56396–405. doi: 10.1021/acsomega.6c07393 (PMC13613464; doi:10.1021/acsomega.6c07393)
Supplement: Supplementary file 1 [file ao6c07393_si_001.pdf]

Supporting Information

In Silico Structure Reactivity Analysis of Lignin-Derived Phenolics: O–H Bond Dissociation Enthalpies, Spin Delocalization, and a Propagator/Terminator Framework for Antifungal-Relevant Activity

Samir Mallick*
Department of Chemistry, University of Pittsburgh, Pittsburgh, Pennsylvania 15260, United States
*E-mail: sam1202@pitt.edu | ORCID: 0009-0004-2428-9774

Contents. Table S1 (GC-MS composition, corncob); Table S2 (GC-MS composition, *Areca catechu*); Figure S1 (phenoxyl-radical spin-density isosurfaces); Figure S2 (VT-1161 redocking validation); Figures S3 and S4 (GC-MS total-ion chromatograms); Table S3 (oxygen spin populations); Table S4 (electronic energies and thermochemistry); Table S5 (O–H BDE summary of the natural phenolics); Section S1 (Cartesian coordinates of optimized geometries).

Table S1. Chemical composition of *Zea mays* corncob wood vinegar determined by GC-MS. Phenolic constituents evaluated in the in silico study are shown in **bold**.

SL	Ret. time (min)	Compound name
1	3.033	2-Furanethanol, β-methoxy-(S)-
2	3.075	3-Methylcyclopent-2-enone
3	3.142	**Phenol**
4	3.350	(Tetrahydrofuran-2-yl)methanol
5	3.658	2-Cyclopenten-1-one, 2,3-dimethyl-
6	3.742	Phenol, 2-methyl-
7	3.917	Phenol, 3-methyl-
8	4.000	3-Ethyl-2-cyclopenten-1-one
9	4.125	**o-Methoxyphenol (guaiacol)**
10	4.417	3-Ethyl-2-hydroxycyclopent-2-en-1-one
11	4.675	Phenol, 2,5-dimethyl-
12	4.858	Phenol, 4-ethyl-
13	5.117	Tridec-1-ene
14	5.217	**Creosol**
15	6.250	**2-Methoxy-4-ethylphenol (4-ethylguaiacol)**

SL	Ret. time (min)	Compound name
16	7.125	**Phenol, 2,6-dimethoxy- (syringol)**
17	7.567	Pentadec-1-ene
18	9.133	**2,4-Di-tert-butylphenol**
19	9.242	Dodecanoic acid, methyl ester
20	10.083	Hexadecanol-1
21	11.650	Tetradecanoic acid, methyl ester
22	12.417	Nonadec-1-ene
23	13.858	Hexadecanoic acid, methyl ester
24	14.558	Ethyl 14-methyl-hexadecanoate
25	15.650	(E)-9-Octadecenoic acid, methyl ester

Table S2. Chemical composition of *Areca catechu* stem wood vinegar determined by GC-MS. Phenolic constituents evaluated in the in silico study are shown in **bold**.

SL	Ret. time (min)	Compound name
1	3.042	5-Methylfuran-2-carbaldehyde
2	3.092	3-Methyl-2-cyclopenten-1-one
3	3.150	Carbamic acid, phenyl ester
4	3.358	(Tetrahydrofuran-2-yl)methanol
5	3.442	p-Benzoquinone, 2-methyl-
6	3.550	3-Methylcyclopentane-1,2-dione
7	3.667	2,3-Dimethylcyclopent-2-en-1-one
8	3.742	Phenol, 2-methyl-
9	3.825	Urocanic acid
10	3.925	Phenol, 3-methyl-
11	4.000	3-Ethyl-2-hydroxycyclopent-2-en-1-one
12	4.125	**Phenol, 2-methoxy- (guaiacol)**
13	4.683	Phenol, 2,5-dimethyl-
14	5.117	(Z)-7-Hexadecene
15	5.217	Phenol, 3-ethoxy-

SL	Ret. time (min)	Compound name
16	5.400	1,4:3,6-Dianhydro-α-D-glucopyranose
17	5.967	**1,2-Benzenediol, 3-methyl- (3-methylcatechol)**
18	6.050	**1,4-Benzenediol, 2-methoxy- (methoxyhydroquinone)**
19	6.250	**Phenol, 4-ethyl-2-methoxy- (4-ethylguaiacol)**
20	7.125	**Phenol, 2,6-dimethoxy- (syringol)**
21	7.575	Tetradecene-1
22	9.133	**2,4-Di-tert-butylphenol**
23	9.433	**2-Propanone, 1-(4-hydroxy-3-methoxyphenyl)- (guaiacylacetone)**
24	10.083	Hexadecanol-1
25	12.417	Nonadecene-1

Figure S1. Hirshfeld spin-density isosurfaces (±0.004 a.u.; blue, positive spin; red, negative spin) of the phenoxyl radicals. For the two benzenediols both candidate radical sites are shown, with the operative (lower-BDE) site indicated. Phenol shows the most oxygen-localized spin and methoxyhydroquinone (para) the most delocalized, consistent with the BDE ordering in the main text.

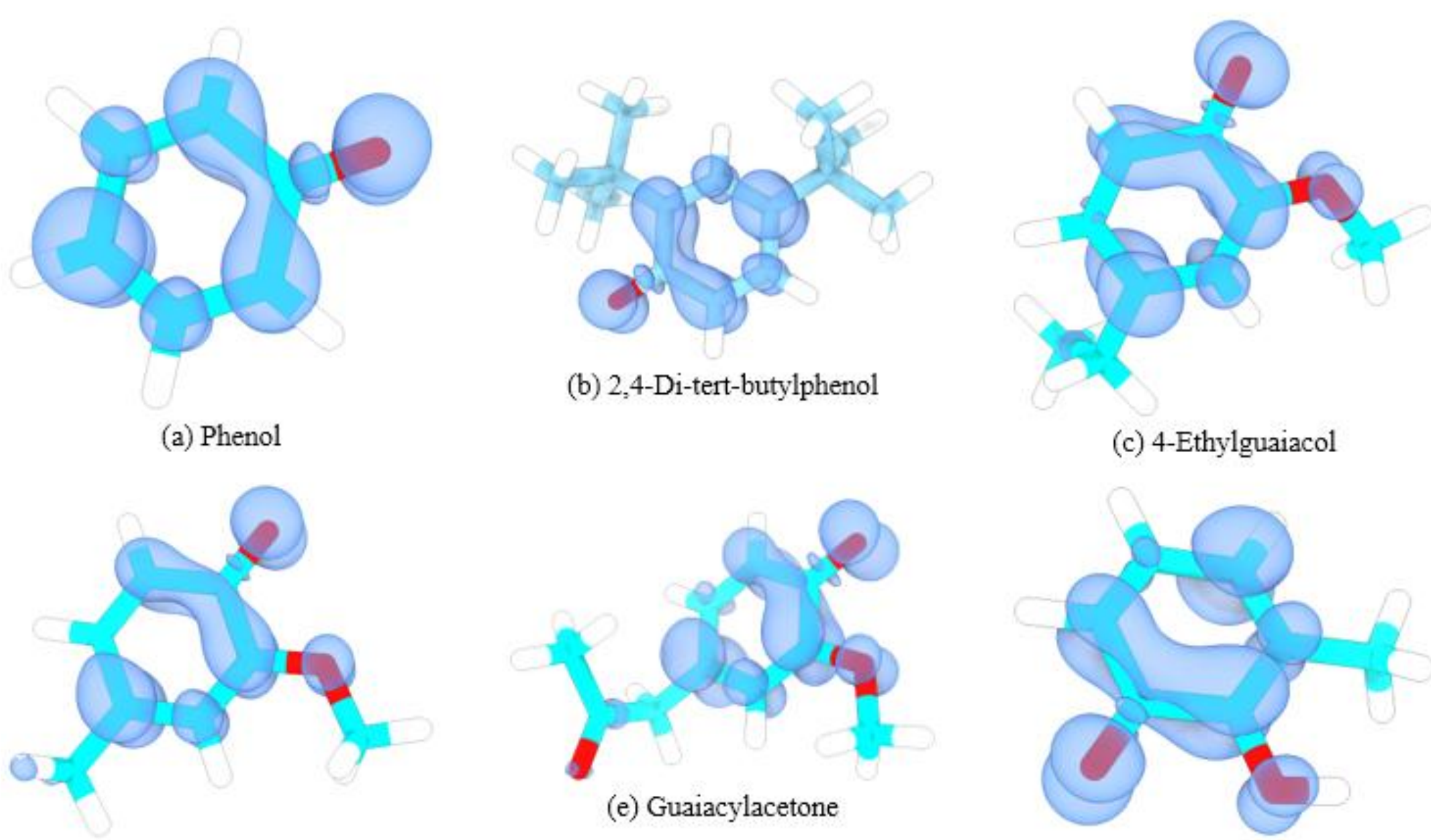

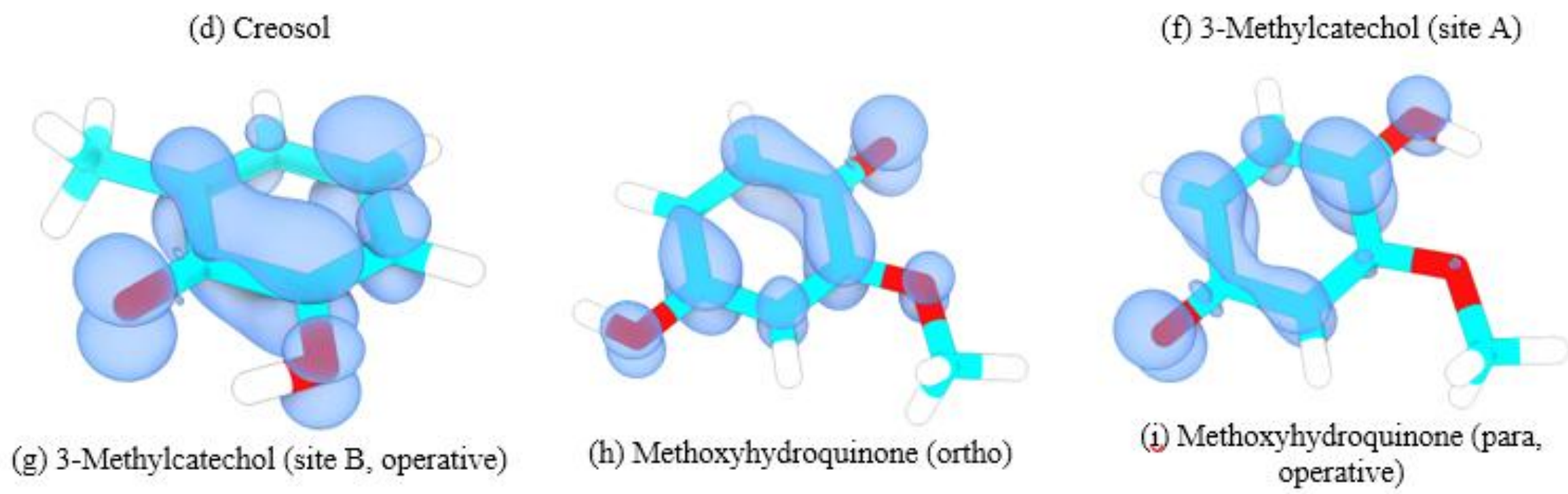

Figure S2. Redocking validation against *C. albicans* CYP51 (PDB 5TZ1). The AutoDock Vina pose of VT-1161 (cyan) overlaid on the crystallographic conformation, reproducing the binding mode (heavy-atom RMSD < 2 Å) at −12.2 kcal mol^{-1}.

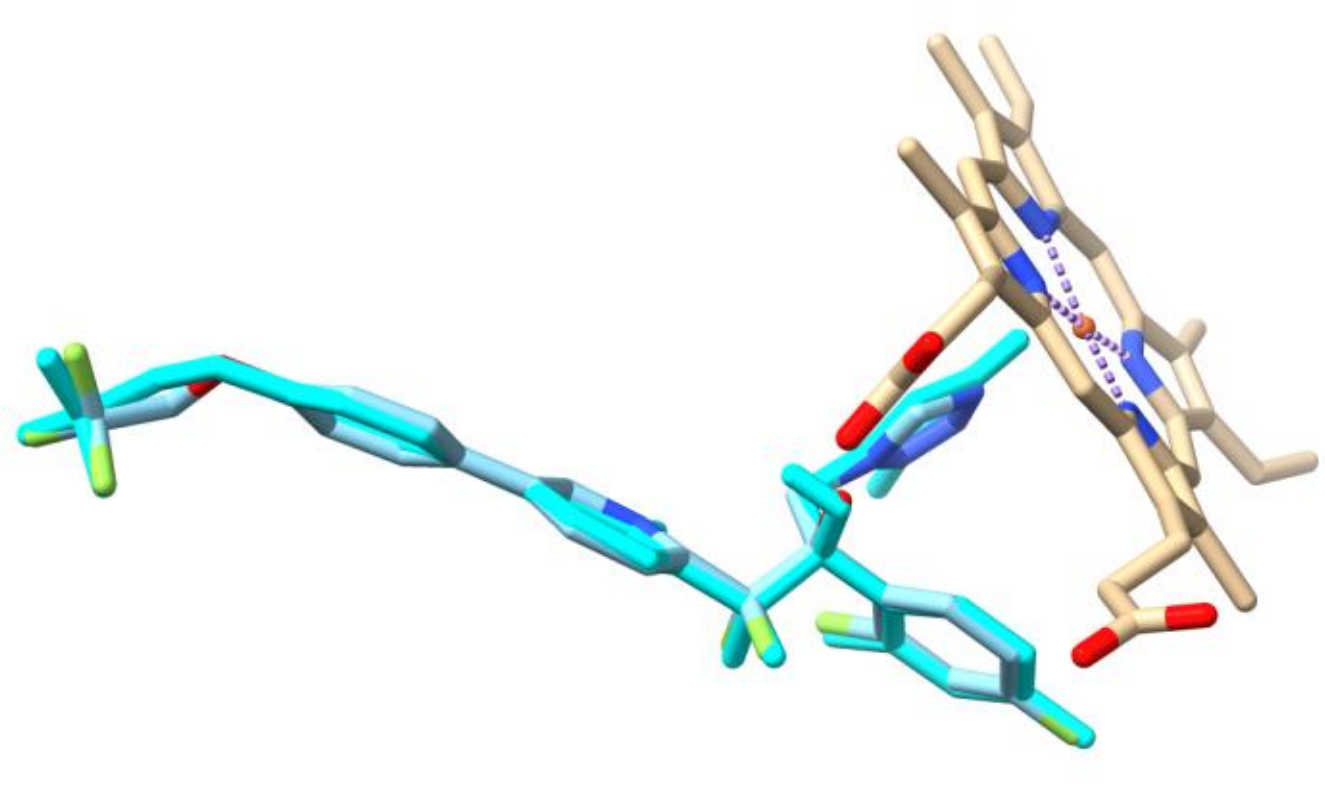

Figure S3. GC-MS total-ion chromatogram (TIC) of corncob (*Zea mays*) wood vinegar. Peaks are labeled with retention times (min); assignments in Table S1.

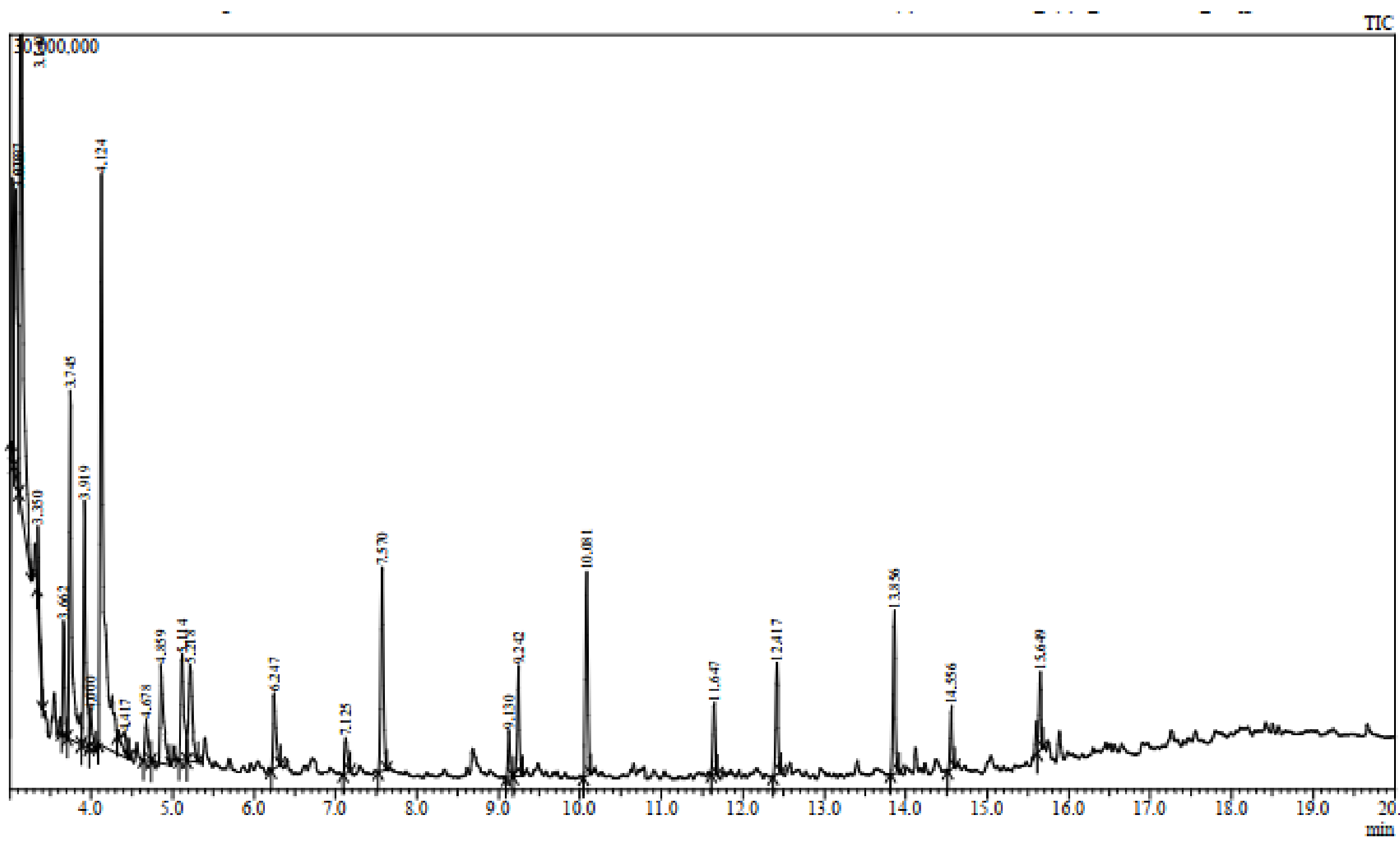

Figure S4. GC-MS total-ion chromatogram (TIC) of *Areca catechu* wood vinegar. Peaks are labeled with retention times (min); assignments in Table S2.

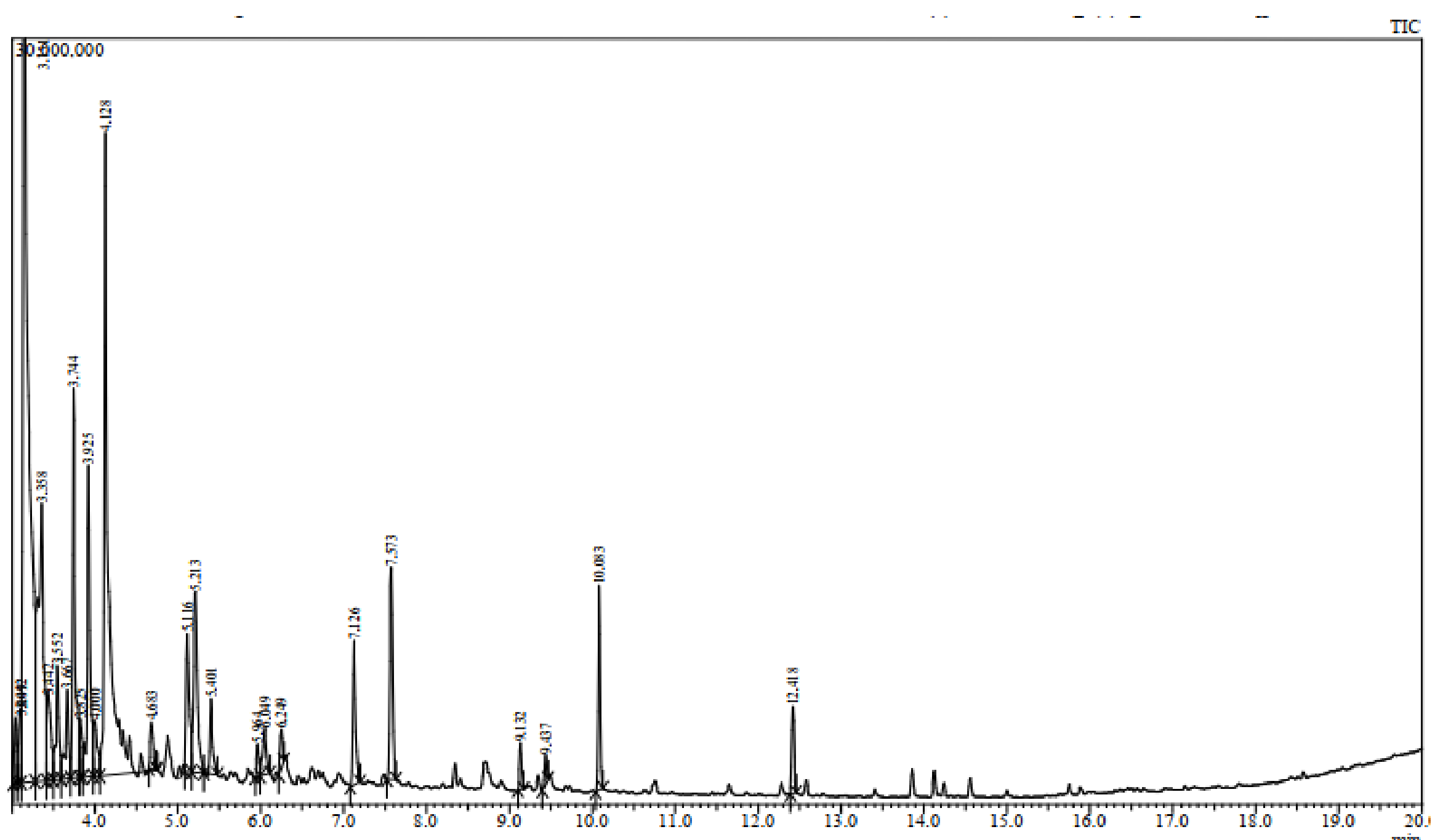

Table S3. Oxygen spin populations of the phenoxyl radicals of the nine natural phenolics (Hirshfeld, Löwdin, Mulliken), ordered by ascending first O–H BDE. The Hirshfeld O-spin

correlates with the first O–H BDE (Pearson r = 0.911), more strongly than Löwdin (0.842) or Mulliken (0.856).

Compound (phenoxyl radical)	Hirshfeld	Löwdin	Mulliken	2nd-highest atom (Löwdin)
Methoxyhydroquinone	0.278	0.285	0.321	C6 (0.182)
3-Methylcatechol	0.294	0.282	0.318	C5 (0.218)
Syringol	0.297	0.285	0.323	C6 (0.210)
Creosol	0.296	0.284	0.321	C1 (0.205)
4-Ethylguaiacol	0.297	0.285	0.322	C2 (0.204)
2,4-Di-tert-butylphenol	0.309	0.307	0.345	C4 (0.240)
Guaiacol	0.302	0.290	0.328	C4 (0.227)
Guaiacylacetone	0.295	0.283	0.321	C4 (0.204)
Phenol	0.352	0.338	0.380	C2 (0.273)

Table S4. Electronic (SCF) energies, zero-point energies, thermal corrections to the enthalpy, 298.15 K enthalpies, imaginary-frequency counts, and ⟨S²⟩ (open-shell species) for all parent species and for the substituted-phenol and peroxyl radicals (B3LYP-D3BJ/def2-TZVP/CPCM(water)). Energies in hartree (Eh).

Species	E_SCF (Eh)	ZPE (Eh)	Thermal corr. (Eh)	H(298 K) (Eh)	# imag.	⟨S²⟩
Phenol	-307.439513	0.104282	0.109760	-307.328808	0	n/a
Guaiacol	-421.955070	0.136674	0.144691	-421.809435	0	n/a
Creosol	-461.264609	0.163734	0.173714	-461.089951	0	n/a
4-Ethylguaiacol	-500.568704	0.192398	0.203555	-500.364204	0	n/a
Syringol	-536.473012	0.168805	0.179600	-536.292469	0	n/a
Guaiacylacetone	-613.902381	0.201448	0.214675	-613.686761	0	n/a
2,4-Di-tert-butylphenol	-621.871881	0.327770	0.344411	-621.526526	0	n/a
3-Methylcatechol	-421.977668	0.135472	0.143996	-421.832728	0	n/a
Methoxyhydroquinone	-497.187970	0.140345	0.149768	-497.037257	0	n/a
4-Aminophenol				-362.668440	0	n/a
4-Aminophenol — phenoxyl radical	-362.175793	0.108412	0.115243	-362.059607	0	0.7622
4-Methoxyphenol	-421.956452	0.136449	0.144584	-421.810924	0	n/a

Supporting Information

Species	E_SCF (Eh)	ZPE (Eh)	Thermal corr. (Eh)	H(298 K) (Eh)	# imag.	⟨S²⟩
4-Methoxyphenol — phenoxyl radical	-421.325703	0.124476	0.132223	-421.192536	0	0.7686
Hydroquinone (4-OH)	-382.669433	0.108217	0.114938	-382.553552	0	n/a
Hydroquinone (4-OH) — phenoxyl radical	-382.038357	0.096227	0.102515	-381.934897	0	0.7697
4-tert-Butylphenol	-464.658322	0.215998	0.226975	-464.430402	0	n/a
4-tert-Butylphenol — phenoxyl radical	-464.020239	0.203419	0.214183	-463.805112	0	0.7773
4-Chlorophenol	-767.006657	0.094848	0.101502	-766.904211	0	n/a
4-Chlorophenol — phenoxyl radical	-766.365439	0.082068	0.088487	-766.276007	0	0.7792
4-Bromophenol	-2880.887702	0.094290	0.101178	-2880.785580	0	n/a
4-Bromophenol — phenoxyl radical	-2880.245798	0.081465	0.088122	-2880.156731	0	0.7794
4-Nitrophenol	-511.961001	0.107007	0.114896	-511.845160	0	n/a
4-Nitrophenol — phenoxyl radical					0	n/a
3-Chlorophenol	-767.007175	0.094783	0.101415	-766.904816	0	n/a
3-Chlorophenol — phenoxyl radical	-766.362349	0.081744	0.088186	-766.273218	0	0.7843
3-Nitrophenol	-511.956856	0.106760	0.114729	-511.841183	0	n/a
3-Nitrophenol — phenoxyl radical	-511.307938	0.093568	0.101396	-511.205597	0	0.7858
3-Methylphenol (m-cresol)	-346.747264	0.131334	0.138692	-346.607628	0	n/a
3-Methylphenol (m-cresol) — phenoxyl radical	-346.106360	0.118639	0.125669	-345.979747	0	0.7817

Species	E_SCF (Eh)	ZPE (Eh)	Thermal corr. (Eh)	H(298 K) (Eh)	# imag.	$\langle S^2 \rangle$
2-Methylphenol (o-cresol)	-346.747480	0.131701	0.138822	-346.607713	0	n/a
2-Methylphenol (o-cresol) — phenoxyl radical	-346.108444	0.118863	0.125804	-345.981696	0	0.7795
2-Chlorophenol	-767.005042	0.094813	0.101492	-766.902606	0	n/a
2-Chlorophenol — phenoxyl radical	-766.364221	0.082070	0.088488	-766.274789	0	0.7800
tert-Butyl hydroperoxide (ROO–H ref)	-308.782226	0.137849	0.145852	-308.635430	0	n/a
tert-butylperoxyl radical (ROO•)	-308.146175	0.126148	0.133748	-308.011483	0	0.7544

Table S5. O–H bond dissociation enthalpies of the nine natural phenolics (first and, for the benzenediols, second O–H BDE in kcal mol^{-1}), $\langle S^2 \rangle$ of the phenoxyl radical, and mechanistic role, ordered by ascending first BDE. Second-BDE products are 3-methyl-1,2-benzoquinone and 2-methoxy-1,4-benzoquinone.

Compound	1st O–H BDE	2nd O–H BDE	$\langle S^2 \rangle$	Role
Methoxyhydroquinone	73.7	61.0	0.77	Propagator
3-Methylcatechol	73.9	69.2	0.77	Propagator
Syringol	74.5	–	0.77	Terminator
Creosol	76.6	–	0.77	Terminator
4-Ethylguaiacol	76.8	–	0.77	Terminator
2,4-Di-tert-butylphenol	77.1	–	0.78	Terminator
Guaiacol	77.3	–	0.77	Terminator
Guaiacylacetone	77.9	–	0.77	Terminator
Phenol	83.2	–	0.78	Terminator

Section S1. Cartesian coordinates of optimized geometries (Å), for all parent species and the substituted-phenol/peroxyl radicals, in XYZ format (species, atom count, then element and x/y/z).

3-Methylcatechol

17

C	0.97497000	-0.07829100	0.03574600
C	2.47657500	-0.07012900	0.12949300
C	3.19029200	-1.26750600	0.08345500
C	4.58654100	-1.27932700	0.16821000
C	5.28427900	-0.08738600	0.29973600
C	4.58411500	1.11430100	0.34707000
C	3.19698600	1.11982200	0.26315000
O	5.27137300	-2.46136700	0.12182100
O	2.60299400	-2.50093700	-0.04825600
H	0.52268600	-0.64700300	0.85435800
H	0.63226000	-0.52859300	-0.90105100
H	0.58455300	0.93719500	0.08076500
H	6.36462200	-0.11501400	0.36439100
H	5.12542900	2.04592500	0.45010000
H	2.65416500	2.05626300	0.30040900
H	4.63459000	-3.18684900	0.04341900
H	1.64063900	-2.42681900	-0.06178700

4-Ethylguaiacol

23

C	-4.41994900	2.37417300	0.03908700
C	-2.91994600	2.23305400	-0.24802500
C	-2.63884600	1.76288600	-1.65321700
C	-2.72594700	0.40010400	-1.96695200
C	-2.51358500	-0.04377600	-3.26407000
C	-2.20780100	0.87751400	-4.27977700
C	-2.11937100	2.22402400	-3.97630100
C	-2.33408300	2.66218400	-2.66890800
O	-1.99234800	0.44658100	-5.55935700

O	-2.56768700	-1.34341000	-3.67922800
C	-2.85313900	-2.35098400	-2.71005700
H	-4.59098200	2.71608000	1.06234700
H	-4.93180100	1.41788300	-0.08938600
H	-4.87757100	3.09467100	-0.64252100
H	-2.48379100	1.52978200	0.46671000
H	-2.42989000	3.19603700	-0.08601200
H	-2.96005000	-0.30849200	-1.18420300
H	-1.87807500	2.92469000	-4.76607900
H	-2.25977700	3.71950000	-2.44474000
H	-2.09443800	-0.51698900	-5.57586400
H	-2.84042800	-3.29550800	-3.24753900
H	-3.83719000	-2.19271000	-2.26318900
H	-2.09285000	-2.36107400	-1.92578300

Phenol

13

C	-1.88587300	0.03712300	-0.00367400
C	-1.20402900	-1.17595700	-0.00353300
C	0.18654200	-1.20676100	0.00178200
C	0.90348100	-0.01127100	0.00690700
C	0.22941200	1.20911500	0.00687200
C	-1.16001600	1.22611600	0.00157200
O	2.26935300	0.01946800	0.01214000
H	-2.96783100	0.05640800	-0.00784300
H	-1.75407000	-2.10889200	-0.00758000
H	0.71778400	-2.15183100	0.00189600
H	0.79933000	2.12964700	0.01091300
H	-1.67799500	2.17727400	0.00152200
H	2.61809100	-0.88234400	0.01040900

Creosol

20

C	3.02076300	0.07638600	0.05684400
C	1.51522000	0.03943500	0.03366700
C	0.84586200	-1.19222900	0.00092700
C	-0.53960500	-1.24593100	-0.01995400
C	-1.28955300	-0.05690400	-0.00804000
C	-0.63575300	1.16025300	0.02405200
C	0.75992600	1.20594600	0.04475300
O	-2.65593100	-0.10355000	-0.02840700
O	-1.29138900	-2.38467600	-0.05254800
C	-0.61674300	-3.64182200	-0.06818200
H	3.41627500	-0.43759900	0.93688100
H	3.38587000	1.10348600	0.07322600
H	3.44304000	-0.41940700	-0.82111200
H	1.42193500	-2.10747400	-0.00825300
H	-1.22204000	2.07078200	0.03278300
H	1.25788100	2.16748700	0.06982100
H	-2.92765300	-1.03348600	-0.04812300
H	-1.39586000	-4.39902000	-0.09546400
H	-0.00949700	-3.76802600	0.83102200
H	0.01730400	-3.73131000	-0.95320400

2,4-Di-tert-butylphenol

37

C	3.66403300	-0.60755600	0.75675000
C	2.50033800	-0.05181600	-0.08622000
C	1.28495300	-0.98880900	0.04976800
C	2.11321500	1.32149500	0.47542100
C	2.92592900	0.02578000	-1.55574800
C	3.30451500	-1.13965100	-2.22852000

145	C	3.71334600	-1.18567200	-3.56277400
146	C	3.72949500	0.04382100	-4.25118000
147	C	3.35880600	1.21870200	-3.60869700
148	C	2.96279500	1.21480600	-2.27805100
149	O	4.10539600	0.19769500	-5.56323500
150	C	4.13015200	-2.51433400	-4.21745900
151	C	4.01353600	-3.69514500	-3.24061900
152	C	3.21461700	-2.84857000	-5.41583900
153	C	5.60806900	-2.45220700	-4.65986600
154	H	3.37291600	-0.66712600	1.80830800
155	H	4.53939200	0.04111800	0.67968400
156	H	3.95288200	-1.60730400	0.42952900
157	H	0.97421100	-1.05293700	1.09537200
158	H	1.51680100	-1.99709500	-0.29564500
159	H	0.44240700	-0.61406200	-0.53565700
160	H	1.81432700	1.21518400	1.51992100
161	H	1.27446300	1.75753700	-0.07073400
162	H	2.94887500	2.02312600	0.43647800
163	H	3.28244500	-2.06526800	-1.67615500
164	H	3.38550100	2.14286300	-4.17302700
165	H	2.68424600	2.15307500	-1.82033600
166	H	4.28230800	-0.65424400	-5.97407700
167	H	2.98777200	-3.83720200	-2.89670300
168	H	4.65613200	-3.56387000	-2.36896100
169	H	4.32503100	-4.60712800	-3.75219400
170	H	2.17380600	-2.90221900	-5.09176300
171	H	3.49598300	-3.81898800	-5.82926600
172	H	3.26274100	-2.12930000	-6.23486700
173	H	6.25027400	-2.28155700	-3.79392600
174	H	5.81888500	-1.66379300	-5.38318700
175	H	5.89542600	-3.40124100	-5.11729300

Guaiacol

17

C	1.05667600	0.06669900	0.04764100
O	2.48263900	0.04603500	0.03174300
C	3.13651700	1.23978600	0.01797500
C	2.51747500	2.48555700	0.01999500
C	3.27980100	3.65530700	0.00548800
C	4.66391400	3.58456700	-0.01110900
C	5.29138600	2.33892600	-0.01338200
C	4.54291200	1.17171000	0.00074700
O	5.10913300	-0.07169100	-0.00135700
H	0.74433400	-0.97451700	0.05653100
H	0.66099600	0.56235400	-0.84233900
H	0.68105800	0.57161600	0.94106600
H	1.43955400	2.55328900	0.03293100
H	2.77890800	4.61477300	0.00728700
H	5.26208600	4.48640100	-0.02234200
H	6.37323600	2.26505700	-0.02635700
H	6.07182400	0.01618600	-0.01326500

Guaiacylacetone

25

C	3.35515800	0.17673700	1.64831000
C	3.11382200	-0.99999600	0.74194800
O	2.56901500	-2.01381800	1.13785900
C	3.57742300	-0.87139500	-0.70123800
C	5.02272000	-0.45184600	-0.82323900
C	6.03203200	-1.37124900	-0.50941800
C	7.36642800	-1.00044100	-0.57581800
C	7.71253000	0.30692800	-0.96100400

C	6.71715400	1.21500200	-1.27360700
C	5.37660000	0.83595200	-1.20351100
O	9.02379200	0.67785300	-1.03200200
O	8.43047600	-1.80580400	-0.29783400
C	8.17487500	-3.15090000	0.10643700
H	2.80748100	0.05423200	2.58039300
H	3.06767100	1.10666300	1.15386300
H	4.42436800	0.24995700	1.86241900
H	2.93904400	-0.11980400	-1.17506500
H	3.39830400	-1.82872000	-1.19191500
H	5.76196500	-2.37557300	-0.21339400
H	6.99702700	2.21712400	-1.57336200
H	4.60567000	1.55614300	-1.44696200
H	9.57010200	-0.08385600	-0.78540800
H	9.14886500	-3.60233100	0.27483900
H	7.64417400	-3.69668200	-0.67685100
H	7.59118300	-3.17371600	1.02935800

Hydroquinone (4-OH)

14

O	-2.77324000	0.02513000	0.00594300
C	-1.40187100	-0.01980100	0.00405300
C	-0.69168600	-1.21603200	0.00177500
C	0.69997800	-1.20415400	0.00000700
C	1.38965800	0.00399800	0.00055200
O	2.76009700	0.07235500	-0.00098100
C	0.67788600	1.20150600	0.00281400
C	-0.71064100	1.18967500	0.00455700
H	-3.12793200	-0.87382200	0.00442000
H	-1.22330500	-2.16067200	0.00136500
H	1.24749300	-2.13967100	-0.00177700

H	3.13032000	-0.82034800	-0.00292700
H	1.21676200	2.14050300	0.00318800
H	-1.26544400	2.11934800	0.00629600

Hydroquinone (4-OH) — phenoxyl radical

13

O	-2.70652800	-0.01404400	0.00715600
C	-1.44904200	-0.00834800	0.00501400
C	-0.68435700	-1.23636600	0.00247700
C	0.68189800	-1.22462500	0.00024100
C	1.37548500	0.00407700	0.00037000
O	2.71552700	0.06819400	-0.00173100
C	0.67081300	1.22742200	0.00274000
C	-0.69421800	1.22657900	0.00499100
H	-1.23225700	-2.17006200	0.00241600
H	1.24793600	-2.14919100	-0.00163700
H	3.10568200	-0.81946800	-0.00313800
H	1.23581400	2.15088000	0.00273700
H	-1.25174600	2.15459600	0.00685500

3-Chlorophenol

13

O	2.59310800	-1.40567300	-0.00147400
C	1.63236500	-0.44124800	-0.00166100
C	1.93734000	0.91932200	0.00066100
C	0.90702600	1.85109900	0.00027300
C	-0.42533800	1.45107500	-0.00242000
C	-0.69816400	0.08934800	-0.00470500
Cl	-2.36779500	-0.44651900	-0.00812200
C	0.30463700	-0.86694000	-0.00438000
H	3.46977700	-0.99711100	0.00107300

H	2.97269600	1.23851500	0.00273100
H	1.14409800	2.90732500	0.00208800
H	-1.22790600	2.17436100	-0.00273900
H	0.07442000	-1.92312500	-0.00617100

3-Chlorophenol — phenoxyl radical

12

O	2.58097900	-1.33616400	-0.00266700
C	1.66233500	-0.48333500	-0.00250900
C	1.94859500	0.93595900	0.00006000
C	0.92920800	1.85322100	0.00018400
C	-0.40926500	1.43439000	-0.00217500
C	-0.70998100	0.05930000	-0.00467800
Cl	-2.38974900	-0.41909900	-0.00754100
C	0.27166000	-0.88995800	-0.00489900
H	2.98732600	1.23895300	0.00186000
H	1.14491800	2.91353200	0.00210700
H	-1.21151300	2.15880200	-0.00207900
H	0.04718500	-1.94751900	-0.00682800

3-Methylphenol (m-cresol)

16

C	2.52848700	1.18395200	0.00304000
C	1.26119500	0.37168100	-0.02102100
C	1.30706100	-1.02520600	-0.03450900
C	0.13052400	-1.76593700	-0.02485900
C	-1.10808000	-1.13427700	-0.00403900
C	-1.15704300	0.25812500	0.00531000
O	-2.33865500	0.94622700	0.02039900
C	0.01933700	1.00410400	-0.00424000
H	3.34512100	0.65419500	-0.48892400

H	2.83974400	1.38261500	1.03291500
H	2.39154100	2.14795400	-0.48858700
H	2.26523000	-1.52988200	-0.05534400
H	0.17390200	-2.84814100	-0.03738500
H	-2.02728700	-1.70865900	0.00007600
H	-3.07977100	0.32524600	0.02139500
H	-0.04588800	2.08577600	-0.00082400

3-Methylphenol (m-cresol) — phenoxyl radical

15

C	2.54158700	1.18563300	-0.00166700
C	1.25278800	0.41360500	-0.00426200
C	1.28541300	-1.00172700	0.04874800
C	0.11036200	-1.76763500	0.04834800
C	-1.11556500	-1.15487000	-0.00409300
C	-1.20574500	0.28889000	-0.05941200
O	-2.31930900	0.86938900	-0.10801700
C	0.03214600	1.03724700	-0.05687000
H	3.15924000	0.90602200	-0.85852700
H	3.12055900	0.96512700	0.89832100
H	2.35829900	2.25854600	-0.04191800
H	2.24510300	-1.50259300	0.09066700
H	0.18139700	-2.84712700	0.08995400
H	-2.03911500	-1.71949400	-0.00582500
H	-0.04257700	2.11731300	-0.09850800

3-Nitrophenol

15

O	2.25261300	0.03716000	0.01114400
C	0.89530100	0.00210000	0.00544500
C	0.17299100	-1.19300500	0.00040700

C	-1.21608000	-1.16863200	-0.00544300
C	-1.90749300	0.03503500	-0.00642400
C	-1.16257800	1.20717200	-0.00114100
C	0.22438300	1.22047900	0.00483200
N	-1.86832000	2.49425100	-0.00200400
O	-3.09294000	2.48386100	-0.01069000
O	-1.20306400	3.52251300	0.00603200
H	2.61114100	-0.86139900	0.00952500
H	0.70365400	-2.13769000	0.00102700
H	-1.76502200	-2.10072300	-0.00927600
H	-2.98528500	0.06727400	-0.01095800
H	0.77420700	2.14897700	0.00872100

3-Nitrophenol — phenoxyl radical

14

O	2.19986500	-0.00766800	0.01156400
C	0.94656000	0.00388700	0.00628000
C	0.17254600	-1.21761000	0.00096400
C	-1.19886500	-1.18399800	-0.00501900
C	-1.88310200	0.03927300	-0.00624900
C	-1.14851100	1.23125700	-0.00092300
C	0.21956200	1.25563300	0.00532900
N	-1.88347200	2.51174600	-0.00228000
O	-3.10433600	2.46825100	-0.01320800
O	-1.23357800	3.54523000	0.00756900
H	0.71509900	-2.15389800	0.00183100
H	-1.76897100	-2.10311000	-0.00892700
H	-2.96180900	0.07177800	-0.01111200
H	0.77354000	2.18231800	0.00933300

Methoxyhydroquinone

18

C	3.10544300	1.02474000	-0.44385600
O	2.72227500	-0.34706400	-0.54076000
C	3.39688400	-1.26407900	0.20716400
C	2.95591200	-2.58840000	0.04399800
C	3.57057200	-3.60153400	0.75449500
C	4.62166700	-3.32109700	1.62981100
C	5.05097400	-2.01097000	1.78600100
C	4.44090100	-0.97501300	1.07519400
O	6.07618500	-1.66243900	2.62875900
O	1.92509700	-2.87547100	-0.81280400
H	2.44599600	1.56703800	-1.11609200
H	4.14381200	1.15958400	-0.75456500
H	2.97718800	1.39170500	0.57679800
H	3.22503700	-4.61917000	0.62312100
H	5.09809400	-4.12087300	2.18352600
H	4.79342300	0.03661400	1.21350100
H	6.42393900	-2.45391400	3.06073900
H	1.62444800	-2.04638200	-1.21323200

2-Chlorophenol

13

O	0.58055600	-1.99834900	0.00071300
C	-0.26075300	-0.93289800	-0.00038100
C	-1.64918100	-1.06589500	0.01059900
C	-2.46609400	0.05700000	0.00895300
C	-1.90966400	1.33197500	-0.00360500
C	-0.52735500	1.47672100	-0.01463700
C	0.28694500	0.35322600	-0.01307000
Cl	2.02422500	0.54438600	-0.02714100
H	0.07179800	-2.82103300	0.00986500

H	-2.07968500	-2.06052000	0.02031300
H	-3.54102700	-0.06896000	0.01752800
H	-2.54183900	2.20974300	-0.00490300
H	-0.07333600	2.45839100	-0.02451300

2-Chlorophenol — phenoxyl radical

12

O	0.51755600	-1.97474900	0.00327900
C	-0.21849600	-0.96883200	0.00081700
C	-1.66304300	-1.07813700	0.01164900
C	-2.46348800	0.02922400	0.00925900
C	-1.89227600	1.31718100	-0.00397500
C	-0.50463300	1.49141300	-0.01509400
C	0.31863000	0.39168900	-0.01313300
Cl	2.03288900	0.58128100	-0.02712600
H	-2.07319300	-2.07974500	0.02169000
H	-3.54056600	-0.07499600	0.01748400
H	-2.53189100	2.18975400	-0.00566200
H	-0.08124300	2.48642100	-0.02520600

2-Methylphenol (o-cresol)

16

C	-1.75158800	-1.60817900	0.01292800
C	-0.49501400	-0.78493700	0.01424600
C	0.76786900	-1.36915600	0.08151000
C	1.93249800	-0.60491300	0.08242700
C	1.84021700	0.78024200	0.01423400
C	0.59208300	1.39070900	-0.05385800
C	-0.56287500	0.61344800	-0.05362200
O	-1.80923400	1.17449700	-0.11959400
H	-1.51390900	-2.66998800	0.07078000

H	-2.33843800	-1.43483700	-0.89274700
H	-2.39466100	-1.35185900	0.85875600
H	0.83490200	-2.44980100	0.13451400
H	2.89942300	-1.08842000	0.13587100
H	2.73446900	1.39084000	0.01370500
H	0.50660700	2.47041800	-0.10790300
H	-1.73164300	2.13710500	-0.16070300

2-Methylphenol (o-cresol) — phenoxyl radical

15

C	-1.75229900	-1.63813600	0.00383300
C	-0.50390000	-0.82078200	0.00524900
C	0.75062300	-1.38539400	0.09326000
C	1.90863800	-0.59802100	0.09268000
C	1.82898700	0.80527400	0.00170100
C	0.60673900	1.41184400	-0.08753800
C	-0.61432100	0.63382300	-0.09044800
O	-1.73424500	1.19290600	-0.17291100
H	-1.52248400	-2.69990700	0.07969700
H	-2.32780600	-1.46468100	-0.90904900
H	-2.40092800	-1.35583500	0.83705400
H	0.84471200	-2.46241700	0.16460700
H	2.87756800	-1.07531100	0.16318900
H	2.73723800	1.39435400	0.00306100
H	0.50693700	2.48763200	-0.15905800

4-Aminophenol

0

4-Aminophenol — phenoxyl radical

14

N	-2.79279000	0.00693400	0.00226600
C	-1.44627300	0.00496400	0.00456100
C	-0.72461500	-1.21981900	0.00072100
C	0.63931900	-1.22410800	0.00298500
C	1.40652700	0.00090600	0.00921300
O	2.66776800	-0.00097800	0.01131900
C	0.64283600	1.22808000	0.01311800
C	-0.72112200	1.22765500	0.01084700
H	-3.31418800	-0.85454200	-0.00217000
H	-3.31156500	0.86999800	0.00528400
H	-1.28108700	-2.14988900	-0.00400800
H	1.18785900	-2.15812000	0.00012800
H	1.19383300	2.16063600	0.01795100
H	-1.27489100	2.15934300	0.01382600

4-Bromophenol

13

O	-4.04460300	0.04295700	-0.00891900
C	-2.68315400	-0.00492100	0.00264200
C	-1.97789900	-1.20714900	0.00853400
C	-0.58804400	-1.20066900	0.02073900
C	0.08574400	0.01169300	0.02682100
Br	2.00391700	0.02417100	0.04386600
C	-0.60478600	1.21648200	0.02082100
C	-1.99311600	1.20656800	0.00866100
H	-4.40831400	-0.85317400	-0.01090100
H	-2.51352700	-2.14915700	0.00369700
H	-0.04437100	-2.13516300	0.02540300
H	-0.07163300	2.15705600	0.02559100
H	-2.54686300	2.13643500	0.00391800

4-Bromophenol — phenoxyl radical

12

O	-3.98535600	0.00270800	-0.00688000
C	-2.73170600	0.00568400	0.00345500
C	-1.97294400	-1.22787600	0.00974100
C	-0.60491600	-1.21859900	0.02096300
C	0.07384700	0.01274300	0.02644000
Br	1.96269600	0.01751900	0.04190200
C	-0.61112800	1.24066500	0.02071100
C	-1.97916500	1.24304300	0.00952000
H	-2.52405800	-2.15946700	0.00525900
H	-0.04346100	-2.14266500	0.02561300
H	-0.05429300	2.16752600	0.02516800
H	-2.53516700	2.17172600	0.00486500

4-Chlorophenol

13

O	-3.28988200	-0.04872000	-0.00106100
C	-1.92781200	0.00277800	-0.00235400
C	-1.22560000	1.20671400	-0.00303600
C	0.16391700	1.20348200	-0.00446000
C	0.84298200	-0.00628100	-0.00519400
Cl	2.59794200	-0.01313300	-0.00703500
C	0.15378200	-1.21217100	-0.00450000
C	-1.23422700	-1.20661800	-0.00306400
H	-3.65546800	0.84658700	-0.00043900
H	-1.76378300	2.14721900	-0.00246600
H	0.70895800	2.13741200	-0.00500300
H	0.69345700	-2.14924600	-0.00506900
H	-1.78544000	-2.13794800	-0.00250100

4-Methoxyphenol

17

C	1.04444600	0.00300000	-0.04016700
O	2.46874900	-0.01832800	-0.06008400
C	3.12574500	1.18097300	-0.07621100
C	2.49800000	2.42404900	-0.07177000
C	3.26277500	3.59099700	-0.08936500
C	4.64951700	3.52582900	-0.11162400
O	5.44719100	4.64176700	-0.12993500
C	5.27797000	2.28032600	-0.11578300
C	4.52161200	1.12023000	-0.09825800
H	0.72876800	-1.03740700	-0.03094600
H	0.64495600	0.49965000	-0.92847100
H	0.67006600	0.50786900	0.85438300
H	1.42130800	2.50605300	-0.05481500
H	2.76771600	4.55544900	-0.08602400
H	4.89793500	5.43687200	-0.12728600
H	6.35928800	2.22922500	-0.13304700
H	5.00538400	0.15161700	-0.10160200

4-Methoxyphenol — phenoxyl radical

16

C	1.04386400	0.00988300	-0.03988500
O	2.48062100	0.02563000	-0.06295500
C	3.12606100	1.19519700	-0.07777200
C	2.48828100	2.45432200	-0.07267900
C	3.24328900	3.59676100	-0.08855400
C	4.68744900	3.55780700	-0.11059700
O	5.37712600	4.60975000	-0.12518800
C	5.29788700	2.24527900	-0.11514800
C	4.54092200	1.11296500	-0.09932800

H	0.76568700	-1.03956500	-0.02989300
H	0.64342700	0.49566200	-0.93037800
H	0.67223800	0.50523800	0.85773400
H	1.41028700	2.52075700	-0.05630600
H	2.76870200	4.56996200	-0.08487800
H	6.37916400	2.19258400	-0.13158600
H	4.99438500	0.12981400	-0.10263500

4-Nitrophenol

15

O	2.22857400	0.01792400	0.01588900
C	0.88086700	-0.01776300	0.00592700
C	0.16501000	-1.22015100	-0.00395200
C	-1.21555100	-1.19924000	-0.01441400
C	-1.87842100	0.02561800	-0.01476800
C	-1.17549700	1.22969600	-0.00461600
C	0.20387400	1.20734900	0.00574700
N	-3.32698400	0.04810300	-0.02628400
O	-3.93147100	-1.02222500	-0.03610500
O	-3.89775500	1.13678700	-0.02581500
H	2.59435700	-0.87874000	0.01331100
H	0.69702100	-2.16339700	-0.00348900
H	-1.77846000	-2.12017900	-0.02228100
H	-1.70971400	2.16760700	-0.00509000
H	0.77209700	2.12755300	0.01354600

4-Nitrophenol — phenoxyl radical

0

4-tert-Butylphenol

25

C	0.92157800	0.10646700	0.29612900
C	2.43185700	0.07928500	0.03196400
C	3.15477100	-0.07444500	1.38407000
C	2.74510900	-1.14371700	-0.85092800
C	2.92089600	1.35149500	-0.66623400
C	2.07591500	2.40648300	-1.00855900
C	2.55035700	3.55212800	-1.64496000
C	3.89978500	3.66525700	-1.95564000
O	4.42684800	4.76256100	-2.58102100
C	4.76729500	2.62619800	-1.62369500
C	4.27631900	1.49450200	-0.99084700
H	0.62371700	-0.82153300	0.78766200
H	0.35164300	0.19237900	-0.63109300
H	0.64144800	0.93577200	0.94868200
H	2.81454500	-0.98084900	1.89046900
H	2.94470500	0.77950000	2.03178900
H	4.23572800	-0.14565700	1.25522000
H	2.40757600	-2.05889900	-0.35856900
H	3.81527100	-1.23803400	-1.03999200
H	2.23527700	-1.06510800	-1.81371100
H	1.02028200	2.35506700	-0.78367700
H	1.86885800	4.35619600	-1.89978600
H	3.72619100	5.40289500	-2.76371900
H	5.81926300	2.71358100	-1.86553200
H	4.97350400	0.70348700	-0.74611400

4-tert-Butylphenol — phenoxyl radical

24

C	0.92063800	0.11221000	0.29370200
C	2.43001300	0.08974200	0.02558300
C	3.16485800	-0.04169600	1.37705500

C	2.75239400	-1.13847900	-0.85197500
C	2.91826100	1.34569500	-0.68041100
C	2.05739800	2.40911100	-1.02752300
C	2.52231200	3.53140500	-1.66305400
C	3.91804500	3.67332700	-2.00561700
O	4.35519900	4.69758200	-2.58852000
C	4.78593000	2.57526200	-1.64211700
C	4.29345200	1.47059500	-1.00898400
H	0.63335800	-0.81399000	0.79338900
H	0.34743200	0.18493000	-0.63227800
H	0.63818200	0.94384300	0.94169600
H	2.82042200	-0.94204500	1.88967900
H	2.95578400	0.81915600	2.01496300
H	4.24451000	-0.11865300	1.24593300
H	2.40807700	-2.04367900	-0.34782000
H	3.82277500	-1.23896300	-1.03255800
H	2.24573200	-1.06823500	-1.81644200
H	1.00581900	2.34439700	-0.79014500
H	1.85512500	4.34282800	-1.92616800
H	5.83678600	2.66076500	-1.88928100
H	4.97179700	0.66843600	-0.75129100

Syringol

21

C	1.80791100	-1.00551500	-0.53366000
O	1.50656400	0.30336500	-0.05111900
C	2.46524200	1.26953400	-0.15951500
C	2.07331000	2.51409400	0.34321800
C	2.95773200	3.59574900	0.29591900
C	4.22936400	3.42190000	-0.25437800
C	4.60345100	2.17772200	-0.74993300

C	3.73273300	1.09397400	-0.70922600
O	2.48523300	4.76523900	0.80893100
C	3.34639600	5.90193400	0.78343900
O	0.83034300	2.68813500	0.88312200
H	0.91910100	-1.60334300	-0.34992300
H	2.65770900	-1.43167200	0.00404900
H	2.02459800	-0.98452200	-1.60392500
H	4.92284000	4.24806200	-0.29715300
H	5.59061500	2.05103100	-1.17493600
H	4.03732900	0.13405100	-1.09854700
H	2.77980200	6.71361400	1.23294800
H	3.61769000	6.16693200	-0.24130000
H	4.25356000	5.72131700	1.36508300
H	0.36436800	1.83999100	0.82804800

tert-Butyl hydroperoxide (ROO–H ref)

16

C	0.99205000	-0.05023700	-0.01368200
C	2.51017600	0.00505700	0.11426100
C	3.00058800	1.40510800	0.46463500
C	3.01738700	-1.02663600	1.11757100
O	3.13734400	-0.45175600	-1.12067800
O	2.78971300	0.42473100	-2.22111500
H	0.53185600	0.19077800	0.94568600
H	0.67000100	-1.05045700	-0.30964400
H	0.63304400	0.66980700	-0.74947100
H	2.58717000	1.71109700	1.42706700
H	2.68455300	2.12665300	-0.28814500
H	4.08958700	1.42093700	0.53462900
H	2.62192900	-0.79092600	2.10619000
H	4.10705600	-1.01260100	1.16879800

H	2.68752500	-2.02960700	0.84287300
H	2.05069500	-0.05279600	-2.63013300

tert-butylperoxyl radical

15

C	0.96925100	-0.03248100	0.01654000
C	2.48061200	0.02278400	0.15516200
C	2.99354100	1.41548700	0.47744000
C	3.01963200	-1.03239800	1.10437500
O	3.08116900	-0.39566300	-1.16689500
O	2.75255800	0.38002600	-2.16930700
H	0.51583400	0.22265600	0.97523400
H	0.64316000	-1.03471200	-0.26511200
H	0.62341800	0.68110200	-0.73056200
H	2.60665900	1.71942600	1.45094800
H	2.65573000	2.13397800	-0.26861900
H	4.08358800	1.42581200	0.51932800
H	2.64654600	-0.83184400	2.10919400
H	4.10989600	-1.01041900	1.13077300
H	2.68921800	-2.02835200	0.80640200

4-Chlorophenol — phenoxyl radical

12

O	-3.22976100	-0.00829600	-0.00243500
C	-1.97609800	-0.00785600	-0.00322000
C	-1.22047400	1.22791200	-0.00382000
C	0.14689600	1.22204100	-0.00466200
C	0.83040400	-0.00725400	-0.00494000
Cl	2.55576300	-0.00698500	-0.00600300
C	0.14739100	-1.23683900	-0.00437500
C	-1.21995500	-1.24329300	-0.00355800

H	-1.77441800	2.15775100	-0.00359000
H	0.71021800	2.14519000	-0.00512600
H	0.71113300	-2.15973300	-0.00461400
H	-1.77368800	-2.17325000	-0.00312300

Note: full Cartesian coordinates and SCF/ZPE/thermal decompositions are tabulated above for every parent species and for the substituted-phenol radicals. For the nine natural-phenolic phenoxyl radicals and the two quinone (second-BDE) species, the O–H bond dissociation enthalpies and $\langle S^2 \rangle$ *are reported in Table S5; their optimized radical geometries can be provided by the corresponding author on request.*
